# Highly Selective and Irreversible Anion-Exchange in Two-Dimensional Bis(terpyridine)metal(II) Polymer Thin Films

**DOI:** 10.1021/acsami.6c00601

**Published:** 2026-03-31

**Authors:** Kenji Takada, Hiroshi Nishihara

**Affiliations:** Research Institute for Science and Technology, 26413 Tokyo University of Science, 2641 Yamazaki, Noda, Chiba 278-8510, Japan

**Keywords:** Coordination polymers, Redox-active polymers, Thin films, Anion-exchange membranes, Anion-storage membranes, Water remediation, Electrochemistry

## Abstract

Bis­(terpyridine)­metal­(II) polymers are functional coordination polymers characterized by cationic backbones containing exchangeable anions inside. Anion-exchange with functional anions can alter or create new properties through interactions between the cationic polymer backbones and anions. Although considerable research has focused on developing functional materials, the fundamental properties of anion-exchange remain unclear. Investigation into the anions applicable for anion-exchange reactions and those selectively incorporated into the polymers is fundamental for understanding the nature of molecular interactions and developing functional anion-exchange materials for further applications, such as anion separation and wastewater remediation membranes. Here, we report the applicability of the anion-exchange reactions of two-dimensional (2D) bis­(terpyridine)­metal­(II) polymers and their excellent selectivity for perrhenate and organic dye anions. Organic dye anions were specifically exchanged, enabling the detection of organic dyes in artificial seawater. Moreover, the anion-exchange was electrochemically irreversible, as demonstrated by the minimal leaching of organic dye anions during redox cycling. These results underscore the potential of 2D bis­(terpyridine)­metal­(II) polymer thin films as highly selective and robust anion-exchange and anion-storage membranes.

## Graphical abstract

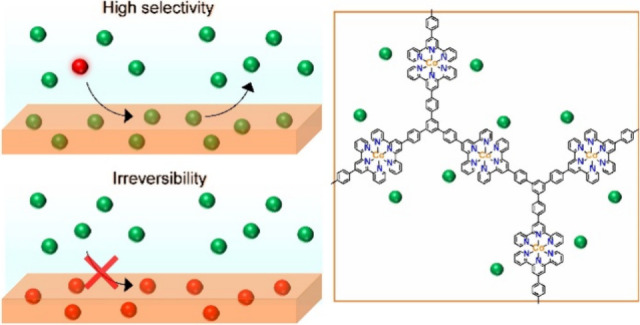

## Introduction

Coordination polymer thin films, including two-dimensional (2D) metal–organic frameworks (MOFs), surface-attached MOFs (surMOFs), and coordination nanosheets, play critical roles in the materials science field,^1–6^ specifically in electronics,^7–11^ photonics,^12–15^ catalysis,^16–19^ and others.^20–23^ Coordination polymers possess characteristics of both organic and inorganic materials, such as high structural and electronic designability, low-weight, flexibility, and facile synthesis under mild conditions. In particular, the structural diversity of coordination polymers arising from a large number of combinations of organic ligands and metal ions endows coordination polymers with various functionalities such as redox activity, magnetism, photophysical properties, electronic properties, and catalytic activities.

In addition to the ligands and metal ions, the choice of counterions is critical for charged coordination polymer films. 2D bis­(terpyridine)­metal­(II) polymers are examples of cationic functional polymers. These materials comprise charge-neutral terpyridine ligands and cationic metal ions; thus, bis­(terpyridine)­metal­(II)-based coordination polymers possess positively charged frameworks, which were the subject of research in the previous studies.^24–35^ In contrast, the anions inside of the cationic framework have not been comprehensively investigated. Accordingly, we have previously modulated photofunctions of bis­(terpyridine)­zinc­(II) polymer thin films (Zn-tpy) by replacing tetrafluoroborate with xanthene dyes,^36^ and, recently, reported the electronic modulation in bis­(terpyridine)­cobalt­(II) polymer thin films (Co-tpy) by anion-exchange from chloride to redox-active metalladithiolene anions.^37^ These studies further motivated us to investigate the postsynthetic functionalization of cationic 2D bis­(terpyridine)­metal­(II) polymer thin films. However, the anion-exchange behavior has yet to be comprehensively understood. Some studies revealed that coordination polymers comprising bis­(terpyridine)­metal­(II) complexes exhibit anion-exchange phenomena and that three-dimensional polymers demonstrate selectivity for organic dye anions.^38–42^ However, the applicability of anion-exchange reactions for a wide variety of anions, along with more detailed insights into their selectivity, remains unexplored. Understanding such fundamentals in anion-exchange reactions is important for the further development of this type of coordination polymer for application in the removal of toxic anions from wastewater or the functionalization of coordination polymers. Furthermore, previously reported anion-exchange materials predominantly exhibit powdery morphologies and mainly focus on spontaneous anion-exchange processes. To fully utilize the redox activity of bis­(terpyridine)­metal­(II) polymers for electrochemical applications, the development of anion-exchange materials with a thin-film morphology is highly desirable.

Here, we demonstrate highly efficient and selective anion-exchange in a two-dimensional bis­(terpyridine)­cobalt­(II)-based coordination polymer thin film (Figure 1). The film was fabricated via a liquid–liquid interfacial coordination reaction between a 3-fold symmetric terpyridine ligand and CoCl_2_, yielding a cationic 2D polymer thin film incorporating chloride counteranions, denoted as Co-tpy (2Cl^–^). Upon immersion of Co-tpy (2Cl^–^) into aqueous solutions containing various anions, spontaneous and quantitative anion-exchange reactions proceeded to form Co-tpy­(*n*X^*m*–^), where X^*m*–^ represents the incorporated anion and *n* and *m* correspond to the number and charge of anions per bis­(terpyridine)­cobalt­(II) unit, respectively. Remarkably, the Co-tpy framework exhibited pronounced selectivity toward inorganic metal oxo-anions and anionic organic dyes, which are representative classes of major wastewater pollutants. Notably, the xanthene dye acid red 91 (AR91^2–^) was selectively captured within the Co-tpy network even in artificial seawater, despite the presence of a high concentration of competing anions. Systematic selectivity studies revealed that interplay of multiple supramolecular host–guest interactions between the anions and the cationic framework plays a more decisive role than simple electrostatic attraction in governing anion uptake. Furthermore, the thin film morphology of the Co-tpy film enabled utilization for electrode materials. Electrochemically driven anion-exchange experiments also demonstrated a strong affinity of the Co-tpy film for organic dye anions, underscoring the robustness of the exchange process. These results establish 2D bis­(terpyridine)­metal­(II) coordination polymer thin films as a versatile platform for highly selective anion adsorption membrane, highlighting their potential not only for the postsynthetic functionalization of coordination polymer films but also for advanced water remediation and separation technologies.

**Figure 1:**
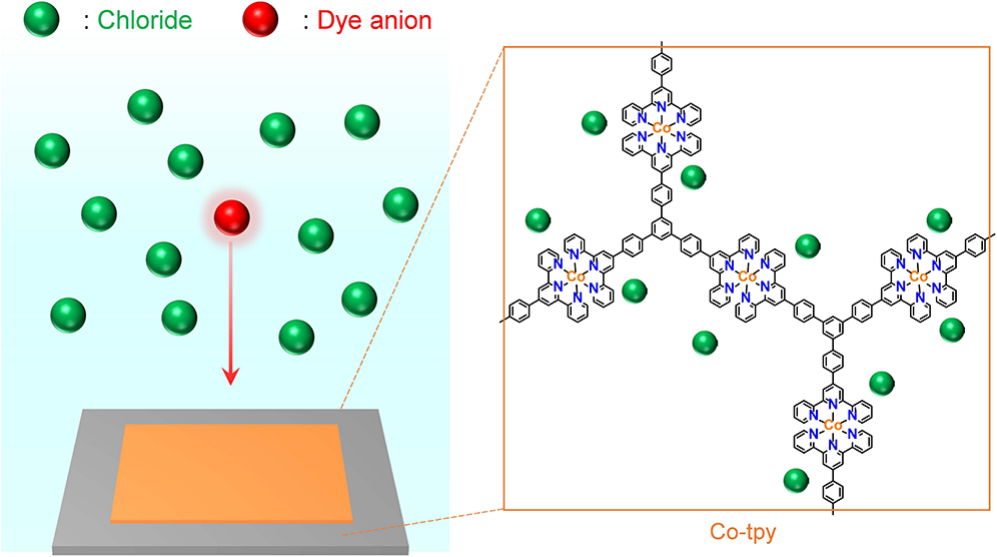
Selective anion-exchange reaction of Co-tpy.

## Results and Discussion

### Anion-Exchange with Inorganic Anions

First, anion-exchange behavior of Co-tpy (2Cl^–^) was investigated using simple halide anions (F^–^, Br^–^, and I^–^). Co-tpy (2Cl^–^) was prepared by the liquid–liquid interfacial coordination protocol using an aqueous CoCl_2_ solution and a CH_2_Cl_2_ solution of the terpyridine ligand, and all characterization results were consistent with previous reports.^27,37^ Co-tpy (2Cl^–^) was obtained as a flat thin film with a thickness of ∼400 nm and a surface roughness of several nanometers.^37^ Anion-exchange was performed by immersing a flake of the Co-tpy (2Cl^–^) film deposited on a substrate (such as silicon, carbon paper, quartz, glass, and others) in aqueous solutions containing 5 mM NaX (X = F, Br, or I) for 3 days (Figure 2a). After 3 days, the shape of the Co-tpy film did not change, suggesting that Co-tpy was not reconstructed. The Raman spectra of Co-tpy (2X^–^) (X^–^ = F^–^, Br^–^, or I^–^) confirmed that the representative peaks of the Co-tpy framework did not change after immersion in the solutions (Figure 2b). Therefore, the Co-tpy framework was stable during the reaction. The anion-exchange efficiency was measured using energy-dispersive X-ray spectroscopy with scanning electron microscopy (SEM-EDS). While the Cl Kα peaks were not detected after the reaction in all cases, the corresponding peaks from the exchanged anions (Br Kα and I Lα) were observed after the reaction (Figure 2c). However, the F Kα peak was not clear owing to overlap with the Co Lα peak. SEM-EDS mapping demonstrated that the constituting elements in the Co-tpy (2X^–^) films were uniformly dispersed (Figures S1–S3). The Na Kα peak was not observed in the SEM-EDS spectra, indicating negligible adsorption of the ion pair. X-ray photoelectron spectroscopy (XPS) also confirmed efficient anion-exchange. Although the Cl 2p peak disappeared after the reaction, the F 1s, Br 3d, and I 3d peaks were detected (Figures 2d and S4–S6). No significant change in the N 1s and Co 2p peak positions indicated the robustness of the bis­(terpyridine)­cobalt­(II) moieties. The results of these spectroscopic studies clearly demonstrate that anion exchange occurred via the simple immersion of Co-tpy (2Cl^–^) into the aqueous solutions containing anions.

**Figure 2:**
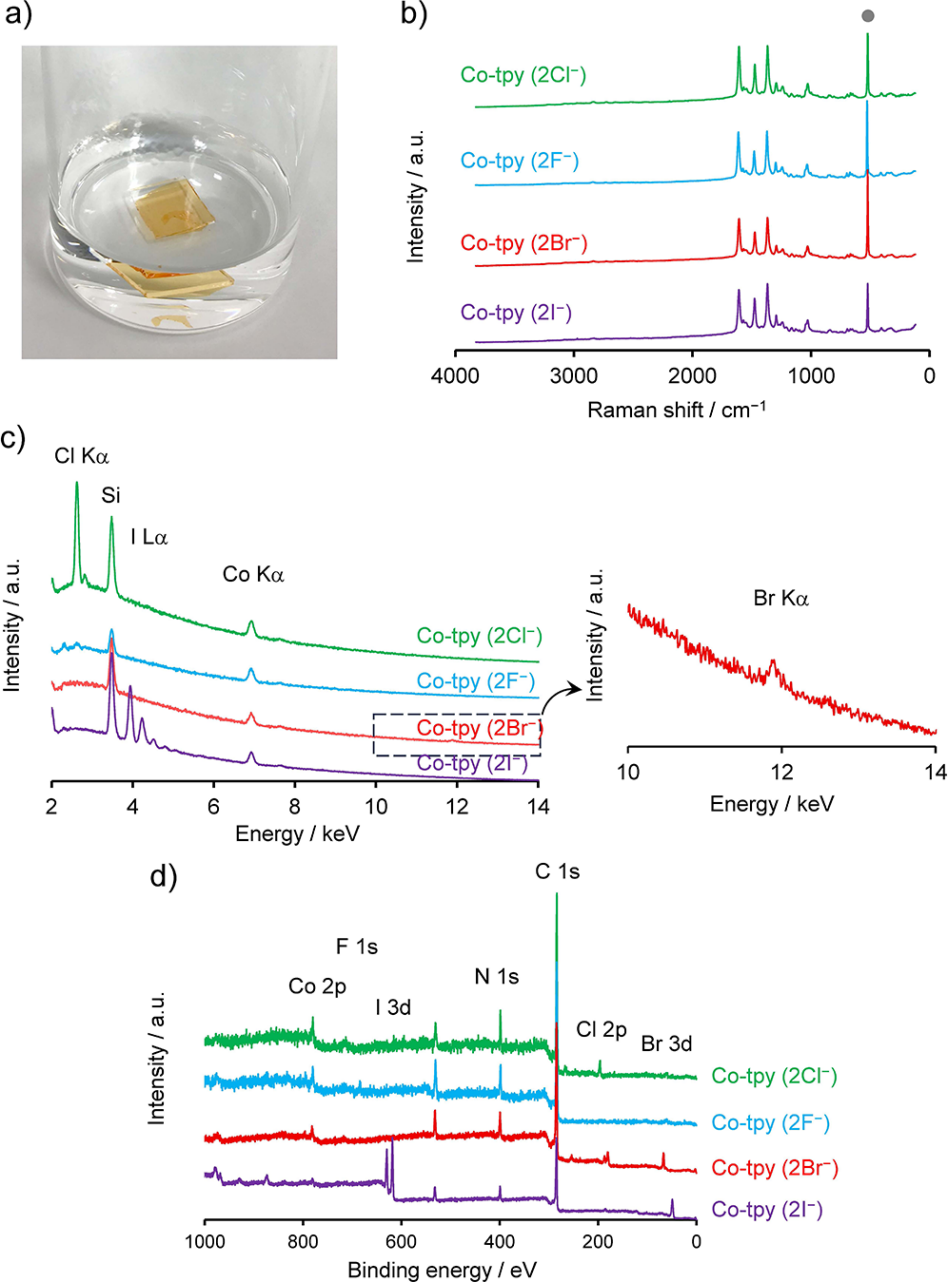
Anion-exchange reaction of Co-tpy. (a) Photographic image showing the typical reaction procedure. Co-tpy decorated on a substrate was immersed in an aqueous solution containing anions. (b) Raman spectra of the as-prepared Co-tpy (2Cl^–^) and anion-exchanged samples. (c) SEM-EDS spectra of the as-prepared Co-tpy (2Cl^–^) and anion-exchanged samples (left) as well as magnification of the Br Kα region (right). (d) XPS survey spectra of the as-prepared Co-tpy (2Cl^–^) and anion-exchanged samples.

The applicability of the anion-exchange reaction was subsequently investigated using multiatomic inorganic anions such as OH^–^, NO_3_^–^, SO_4_^2–^, CF_3_COO^–^, ClO_4_^–^, BF_4_^–^, PF_6_^–^, and SbF_6_^–^. Anion-exchange was confirmed by SEM-EDS (Figure 3a). In all cases except ClO_4_^–^, the Cl Kα peak was not detected after anion-exchange. New peaks from the inserted SO_4_^2–^, CF_3_COO^–^, BF_4_^–^, PF_6_^–^, and SbF_6_^–^ anions were detected. These results suggested the anion-exchange reaction of Co-tpy (2Cl^–^) with multiatomic anions. The XPS results confirmed anion-exchange with NO_3_^–^ and ClO_4_^–^ (Figure 3b,c). Co-tpy (2NO_3_^–^) exhibited a new N 1s peak at 405.7 eV corresponding to the nitrate anion,^31^ while the Cl 2p_3/2_ peak for Co-tpy (2ClO_4_^–^) was observed at 207.1 eV, indicating perchlorate insertion into Co-tpy. Therefore, Cl^–^ ions in Co-tpy (2Cl^–^) can be replaced with various multiatomic inorganic anions.

**Figure 3:**
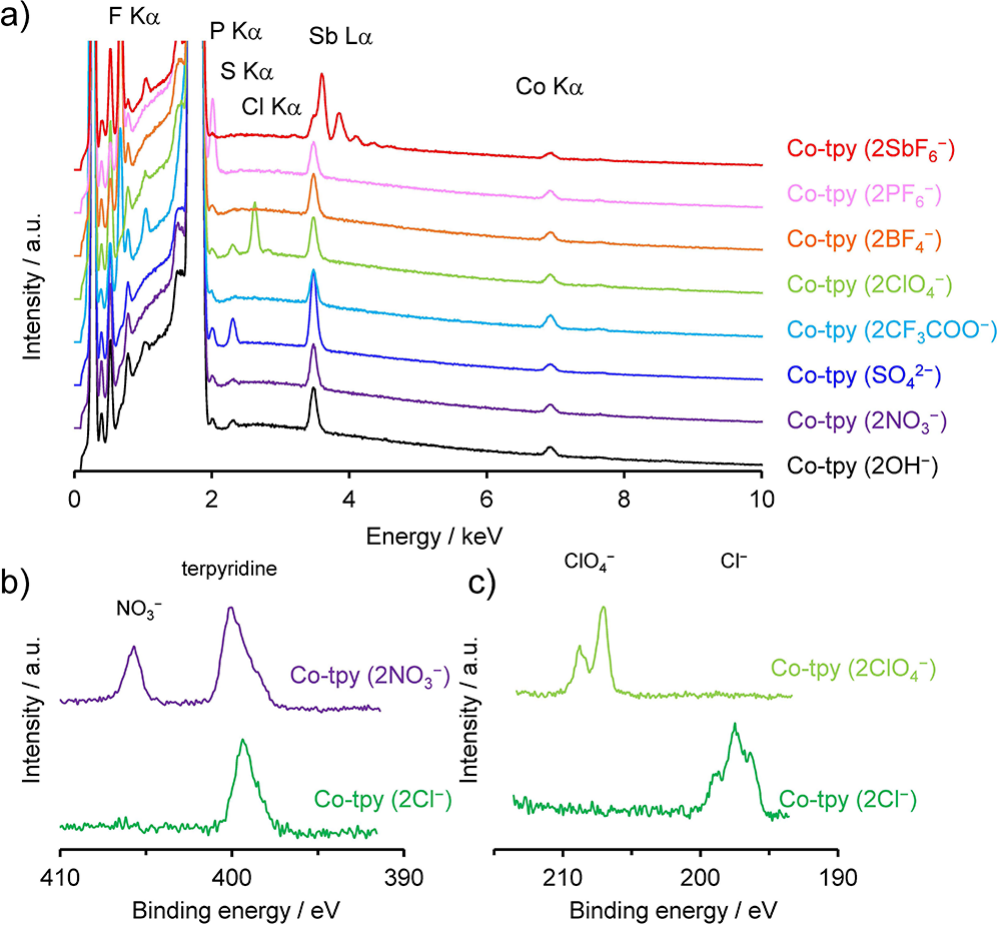
Anion-exchange with multiatomic anions. (a) SEM-EDS spectra of the anion-exchanged Co-tpy. (b) XPS spectra of Co-tpy (2Cl^–^) and Co-tpy (2NO_3_^–^) focusing on the N 1s core level. (c) XPS spectra of Co-tpy before and after anion-exchange with Co-tpy (2Cl^–^) and Co-tpy (2ClO_4_^–^) focusing on the Cl 2p core level.

Electrochemical measurements were performed to gain mechanistic insights into the anion-exchange process. The cyclic voltammograms of Co-tpy (2Br^–^), Co-tpy (2CH_3_COO^–^), and Co-tpy (2NO_3_^–^) exhibited redox waves attributed to the [Co­(tpy)_2_]^2+^/[Co­(tpy)_2_]^+^ redox couple (Figure S7). The redox potentials were −1.13, −1.14, and −1.12 V versus ferrocenium/ferrocene (vs Fc^+^/Fc), respectively, which are comparable to that of Co-tpy (2Cl^–^) (−1.15 V vs Fc^+^/Fc).^25^ These results suggest that the cobalt coordination environment undergoes no significant changes despite the coordination ability of the anions. Therefore, the exchanged anions are accommodated through noncoordinative interactions with the Co-tpy framework rather than direct coordination to cobalt ions.

Cation tolerance was investigated using a series of tetrafluoroborate salts, namely, HBF_4_, *n*Bu_4_NBF_4_, NH_4_BF_4_, and KBF_4_. While Na^+^, K^+^, and *n*Bu_4_N^+^ salts yielded neutral solutions, H^+^ and NH_4_^+^ salts resulted in acidic conditions. Figure S8 shows the SEM-EDS spectra of the Co-tpy film immersed in these tetrafluoroborate salts. The SEM-EDS results indicated that the chloride ion in Co-tpy (2Cl^–^) was quantitatively replaced by BF_4_^–^, yielding Co-tpy (2BF_4_^–^). These results clearly demonstrate that the anion-exchange process is independent of the countercation and proceeds efficiently even under acidic conditions, underscoring the robustness of the Co-tpy framework toward diverse chemical environments.

The anion-exchange strategy offers easy access to Co-tpy encapsulating various anions. For example, although Co-tpy (2NO_3_^–^) thin films can be directly synthesized by the liquid–liquid interfacial coordination reaction between the terpyridine ligand and Co­(NO_3_)_2_, the resulting film thickness (160 nm after 3 days of growth, Figure S9) was markedly thinner than Co-tpy (2NO_3_^–^) obtained via the anion-exchange reaction from Co-tpy (2Cl^–^) (400 nm). The anion-exchange reaction enables Co-tpy films with specific thicknesses that cannot be obtained by direct interfacial coordination reactions. Significantly, the anion-exchange reaction enables the synthesis of Co-tpy (2OH^–^), a compound that cannot be synthesized through the direct interfacial coordination reaction because Co­(OH)_2_ is insoluble in water. This anion-exchange strategy successfully realized Co-tpy with a range of thicknesses and anion compositions, which cannot be realized by direct liquid–liquid interfacial reactions with commercially available cobalt salts.

The reversibility of the anion-exchange reaction was also investigated. The immersion of directly synthesized Co-tpy (2BF_4_^–^) in a 5 mM aqueous NaCl solution yielded Co-tpy (2Cl^–^). SEM-EDS revealed the partial occurrence of anion-exchange from BF_4_^–^ to Cl^–^; however, some BF_4_^–^ remained in the Co-tpy film, where the exchange ratio was estimated to be 90% (Figure S10). This result suggests that the anion-exchange reaction is reversible for such simple inorganic anions; however, the system exhibits a differential affinity for the various anions. Differences in the affinity of the Co-tpy framework for anions can cause selective or even irreversible anion uptake into Co-tpy, as discussed later.

### Anion-Exchange to Organic Dye Anions

Anions were subsequently exchanged with anionic organic dyes of various colors. Figure 4a shows the structures of the anionic dyes used in this study, acid red 91 (AR91^2–^), bromophenol blue (BPB^–^), bromocresol green (BCG^–^), indigo carmine (IC^2–^), eriochrome black T (ET^–^), and tropaeolin O (TO^–^). Anion-exchange experiments were performed using the same method as that used for NaBr: the simple immersion of a Co-tpy (2Cl^–^) film on a substrate in 5 mM aqueous dye solutions. The successful anion-exchange of Co-tpy with ET^–^ required the use of MeOH as a solvent instead of H_2_O, probably with a larger stabilization energy owing to the low solubility of ET^–^ in MeOH. After the anion-exchange reaction, Co-tpy exhibited the colors of the dye anions, indicating the incorporation of the anionic dyes into the Co-tpy framework (Figure 4a). Ultraviolet–visible (UV–vis) spectroscopy confirmed the incorporation of the organic dyes (Figure 4b).^43–47^ SEM-EDS revealed that the chloride ions were efficiently replaced with the dyes (Figure 4c). These results confirm the successful anion-exchange reaction of Co-tpy with organic xanthene and azo dye anions, yielding high anion-exchange capacities of 736–1751 and 528–726 mg/g for monovalent and divalent anions, respectively.

**Figure 4:**
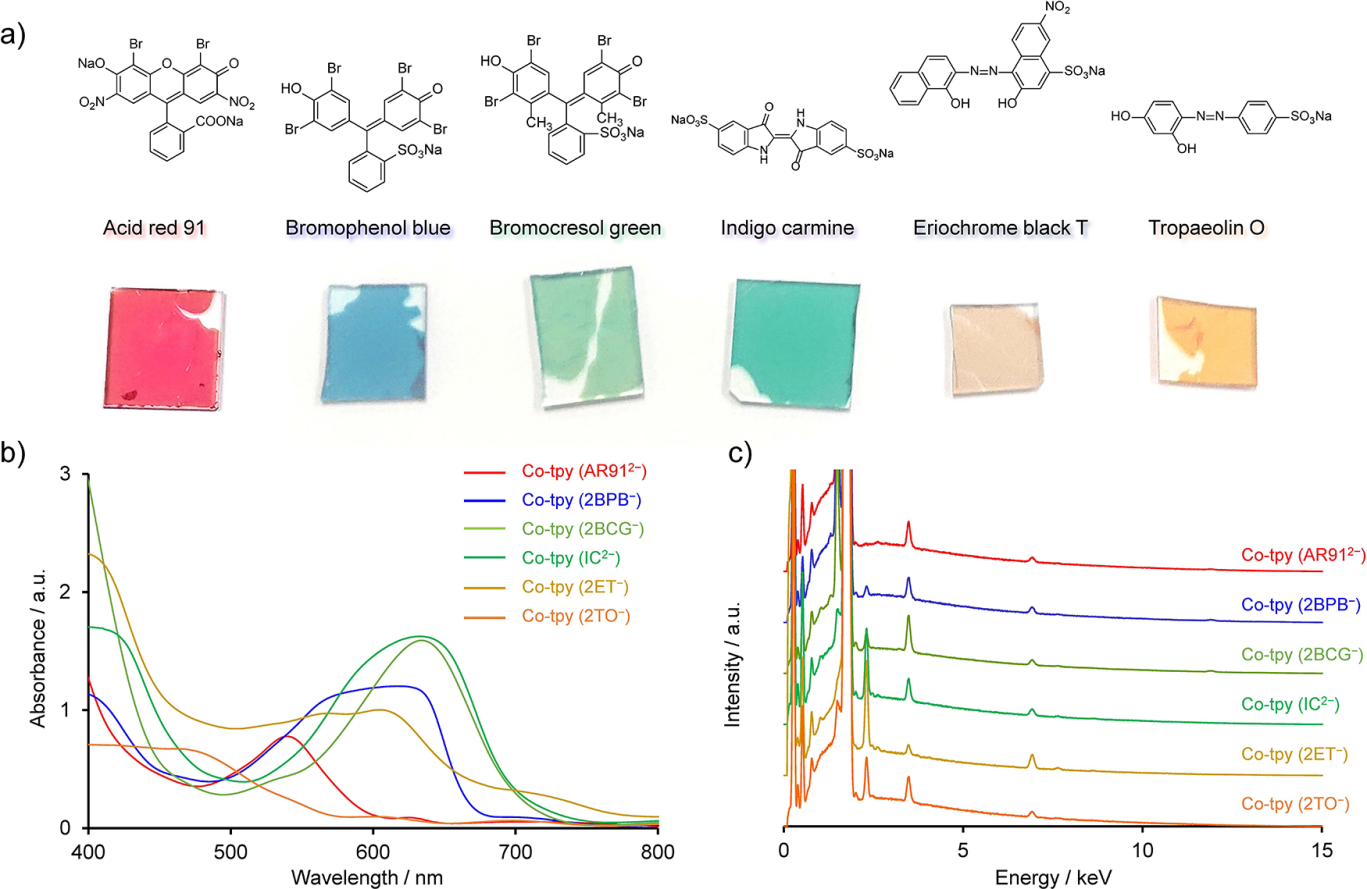
Anion-exchange with organic dye anions. (a) Chemical structures for the sodium salts of the anionic organic dyes used in this study alongside photographic images of the Co-tpy films on glass substrates after anion-exchange with each dye. (b) UV–vis spectra of Co-tpy after anion-exchange with the various dyes. (c) SEM-EDS spectra of Co-tpy after anion-exchange with the various dyes.

Unlike the simple inorganic anions, anion-exchange with organic dyes is irreversible. Further anion-exchange of Co-tpy (AR91^2–^) with sulfate did not proceed. The film was still colorized with AR91^2–^, while the solution was colorless after a three-month reaction with a 5 mM aqueous Na_2_SO_4_ solution (Figure S11a). The UV–vis spectrum also confirmed the absence of AR91^2–^ by immersion in the Na_2_SO_4_ solution (Figure S11b). The irreversibility was attributed to the high selectivity for AR91^2–^ as described in the following sections.

### Anion-Exchange to Metal Oxo-Anions

In addition to the organic pollutants, the anion-exchange capability of Co-tpy (2Cl^–^) toward heavy metal oxoanions (inorganic pollutants) was investigated. Anion-exchange was similarly carried out in a 5 mM aqueous solution for 3 days, and the exchange ratio was investigated by using SEM-EDS. The results confirmed sufficient anion-exchange with ReO_4_^–^, Cr_2_O_7_^2–^, WO_4_^2–^, MoO_4_^2–^, and TeO_3_^2–^ (Figure 5a). Anion-exchange with MnO_4_^–^ with 5 mM aqueous KMnO_4_ led to the decomposition of Co-tpy (2Cl^–^), possibly due to oxidation due to the strong oxidation ability of permanganate. Conversely, the use of a diluted solution of 5 μM resulted in the maintenance of the Co-tpy backbone and facilitated efficient anion exchange (Figure 5a). ReO_4_^–^ and Cr_2_O_7_^2–^ were also efficiently exchanged under the 5 μM diluted condition (Figure S12). XPS analyses revealed that the Cr 2p_3/2_ and Re 4f_7/2_ peaks were located at 579.0 and 45.2 eV, respectively. Therefore, these oxo-anions retained their high oxidation states of +6 and +7 for Cr_2_O_7_^2–^ and ReO_4_^–^, respectively, after encapsulation (Figure 5b,c). Therefore, a wide variety of oxo-anions can be incorporated into the Co-tpy framework. Owing to the stoichiometric anion-exchange reaction, the anion-exchange capacities of ReO_4_^–^, Cr_2_O_7_^2–^, and MnO_4_^–^ were calculated to be 628, 271, and 299 mg/g, respectively. The cyclic voltammogram of Co-tpy (2ReO_4_^–^) showed a redox potential of −1.13 V vs Fc^+^/Fc (Figure S13), consistent with the [Co­(tpy)_2_]^2+^/[Co­(tpy)_2_]^+^ redox couple, confirming that the bis­(terpyridine)­cobalt­(II) coordination sites do not undergo significant coordination-environment changes after anion-exchange.

**Figure 5:**
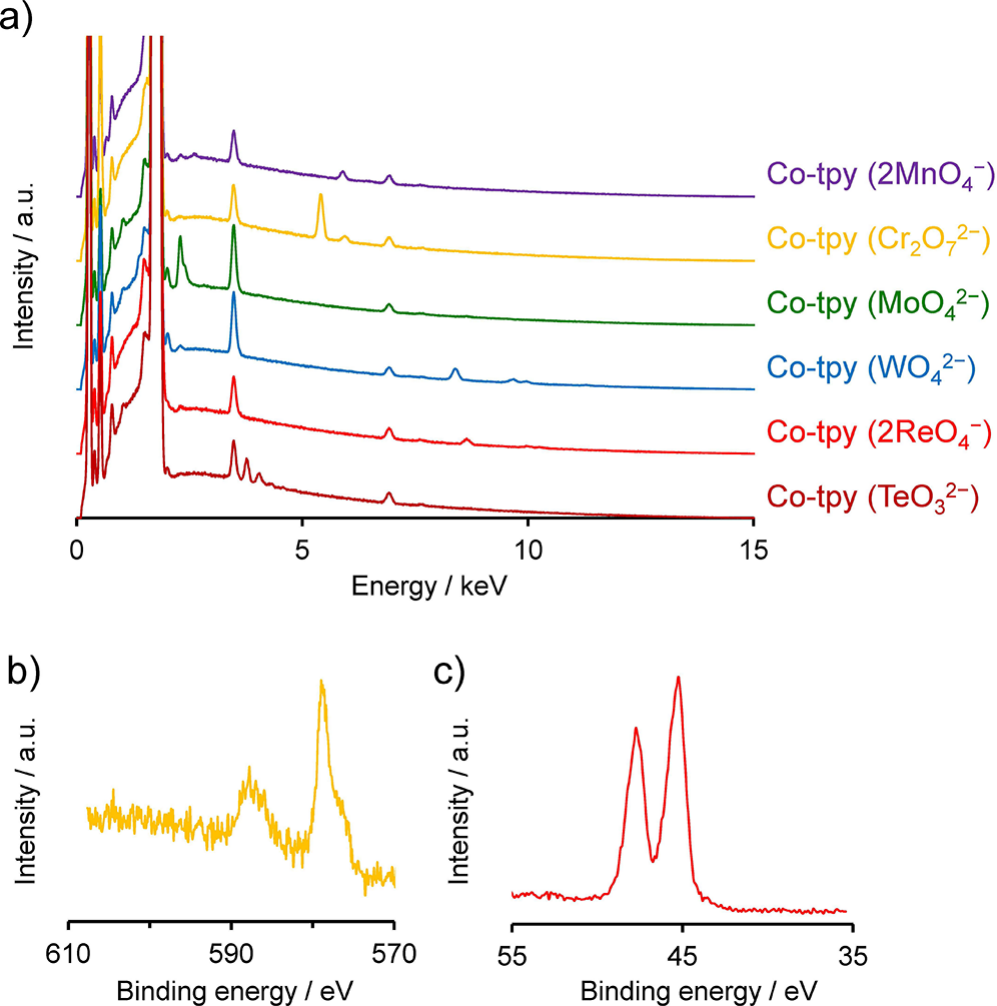
Anion-exchange with oxo-anions. (a) SEM-EDS spectra of Co-tpy anion-exchanged with MnO_4_^–^, Cr_2_O_7_^2–^, MoO_4_^2–^, WO_4_^2–^, ReO_4_^–^, and TeO_3_^2–^. (b) XPS spectrum of Co-tpy (Cr_2_O_7_^2–^) focusing on the Cr 2p core level. (c) XPS spectrum of Co-tpy (2ReO_4_^–^) focusing on the Re 4f core level.

### Kinetic Investigation

The kinetics of the anion-exchange reaction of the organic dyes were investigated using UV–vis spectroscopy. Figure 6a shows the change in the absorption during the anion-exchange reaction with 3 μM AR91^2–^. The solution became colorless after the reaction (Figure 6a). The intensity of the AR91^2–^ peak at 517 nm in the aqueous phase gradually decreased and was approximately zero after 3 days (Figure 6b). The anion-exchange reaction with ReO_4_^–^ demonstrated similar kinetics. UV–vis spectroscopy showed that anion-exchange was complete within 40 h (Figure S14). Anion-exchange is relatively slower than that in other porous polymers such as powdered covalent–organic frameworks (COFs) and MOFs.^48–51^ The linear logarithm plots of the absorbance change indicate first-order reaction kinetics (Figure S15). The sluggish anion-exchange of the Co-tpy film was caused by the thin-film morphology of Co-tpy with a low surface area. The experimental conditions without stirring were also attributed to sluggish anion-exchange. The low crystallinity of the Co-tpy film and lack of regular porosity impeded the mass transport of anions within the Co-tpy films, unlike the smooth anion-exchange observed in crystalline COFs and MOFs possessing regular porosity. The slower anion-exchange observed for AR91^2–^ relative to ReO_4_^–^ indicates that small-sized anions are exchanged more rapidly.

**Figure 6:**
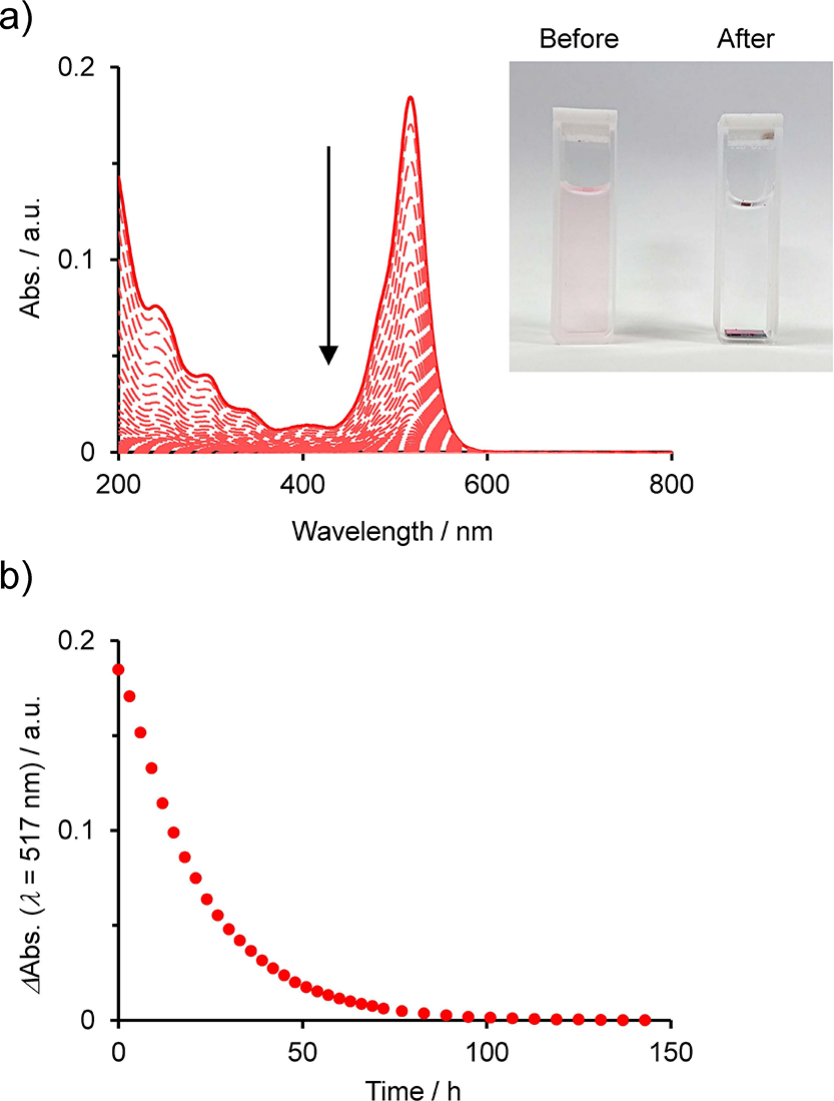
Time-dependent UV–vis spectroscopy. (a) UV–vis spectra of the anion-exchange reaction of Co-tpy with AR91^2–^ in a 3 μM aqueous Na_2_AR91 solution recorded every 3 h over 6 days. The inset shows photographic images of the 3 μM aqueous Na_2_AR91 solution (left) and anion-exchanged solutions (right). (b) Temporal evolution of the absorption change at 517 nm during the anion-exchange reaction.

### Applicability for Other M-tpys

The anion-exchange reaction was also demonstrated in the bis­(terpyridine)­iron­(II) and bis­(terpyridine)­nickel­(II) polymers (Fe-tpy (2BF_4_^–^) and Ni-tpy (2Cl^–^)), which contained BF_4_^–^ and Cl^–^ in the as-prepared states, respectively. Both Fe-tpy and Ni-tpy were synthesized via a liquid–liquid interfacial synthesis. The SEM-EDS indicated that the anions such as Br^–^, SO_4_^2–^, and AR91^2–^ efficiently replaced the anions (Figures S16a and S17a). Raman spectroscopy also confirmed that the anion-exchange reaction did not change the Raman scattering from the M-tpy backbones (Figures S16b and S17b). Furthermore, as mentioned in the Introduction, our group previously reported anion-exchange of Zn-tpy from BF_4_^–^ to xanthene dye anions (ethyl eosin and eosin Y).^36^ Therefore, this facile anion-exchange strategy is also applicable to the postsynthetic functionalization of this series of coordination polymer films.

### Selectivity in Anion-Exchange Reaction with Inorganic Anions

The selectivity of the anion-exchange behavior was investigated by using different anions. First, anion-exchange selectivity between halides was investigated. Anion-exchange was performed in a solution containing equivalent concentrations of halides (F^–^, Cl^–^, Br^–^, and I^–^), and the resultant Co-tpy was investigated using SEM-EDS. The spectrum in Figure 7a suggest that the F^–^:Cl^–^:Br^–^:I^–^ molar ratio was 2:3:17:78. This means that Co-tpy favors a heavier halide. If electrostatic interactions between the cationic Co-tpy framework and halides were dominant, then F^–^ would be the most strongly exchanged anion due to its highest charge density. However, the observed selectivity order is F^–^ < Cl^–^ < Br^–^ < I^–^, indicating that supramolecular interactions, rather than purely electrostatic attraction, play a dominant role in determining selectivity.

**Figure 7:**
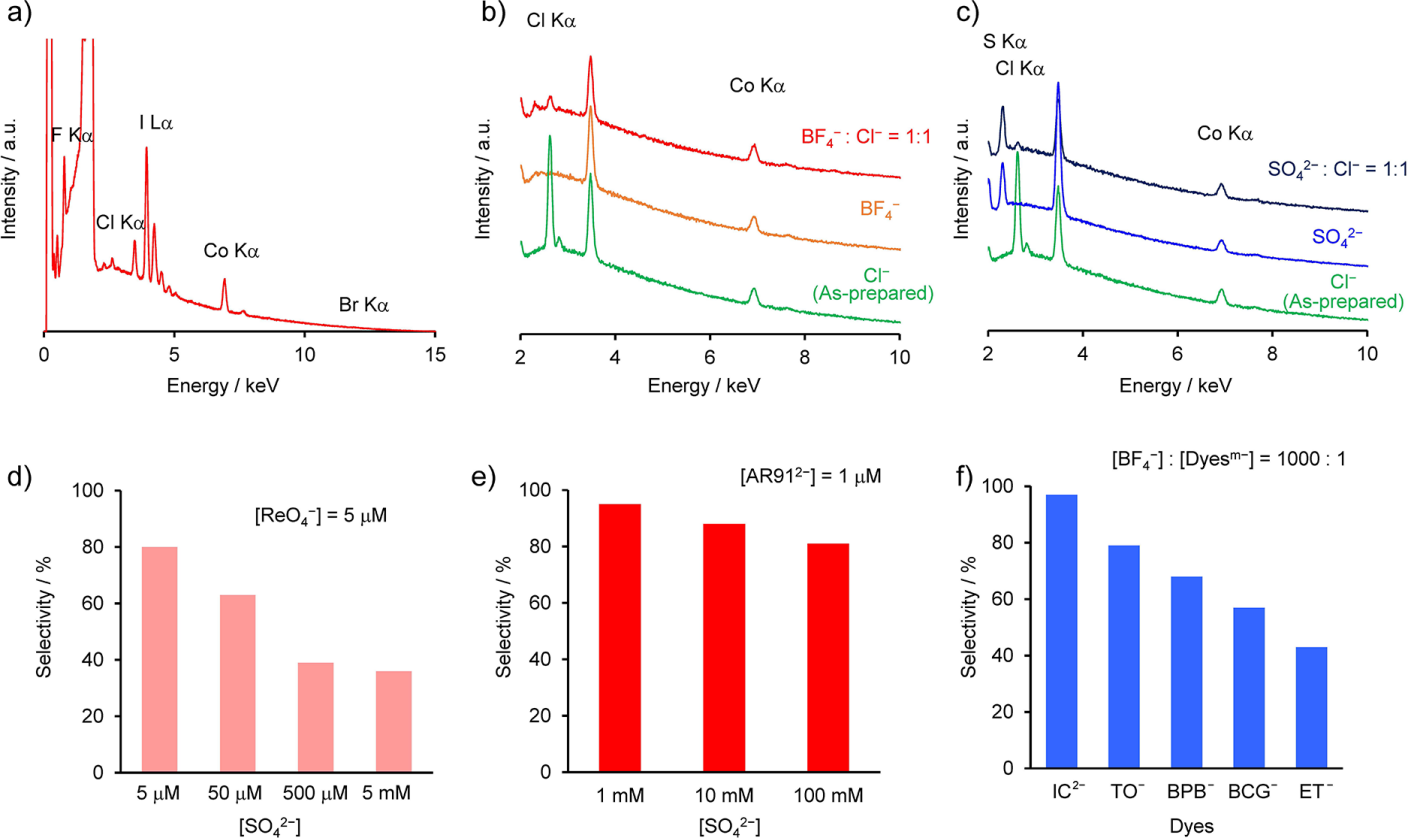
Selective anion-exchange reaction of Co-tpy. (a) SEM-EDS spectrum of Co-tpy after anion-exchange with the halide-mixture solution. (b) SEM-EDS spectra of Co-tpy before (green) and after anion-exchange with BF_4_^–^ (orange) and a mixture solution of BF_4_^–^ and Cl^–^ (red). (c) SEM-EDS spectra of Co-tpy before (green) and after anion-exchange with SO_4_^2–^ (blue) and a mixture solution of SO_4_^2–^ and Cl^–^ (navy). (d) Selectivity for ReO_4_^–^ over SO_4_^2–^ at various molar ratios. (e) Selectivity for AR91^2–^ over SO_4_^2–^ at various molar ratios. (f) Selectivity for organic dyes over BF_4_^–^.

Subsequently, the selectivity for inorganic anions was investigated by using Cl^–^, BF_4_^–^, and SO_4_^2–^. To investigate the selectivity, Co-tpy (2Cl^–^) was immersed in aqueous solutions containing two anions with the same molar concentration (2.5 mM for each anion). Figure 7b shows the respective SEM-EDS spectra. Reaction of Co-tpy with Cl^–^ and BF_4_^–^ resulted in BF_4_^–^ occupying 89% of the total negative charge, as quantified by the Cl Kα peak area. Reaction of Co-tpy with Cl^–^ and SO_4_^2–^ resulted in SO_4_^2–^ occupying 89% of the total negative charge, as quantified by the Cl Kα peak area. These results indicate the superior selectivity for polyatomic anions such as BF_4_^–^ and SO_4_^2–^ over single-atomic Cl^–^. In contrast, the anion-exchange reaction performed in the mixture of BF_4_^–^ and SO_4_^2–^ resulted in 40% SO_4_^2–^ selectivity (Figure S18), thereby indicating that BF_4_^–^ and SO_4_^2–^ exhibited similar affinities to the Co-tpy framework in spite of the charge difference. These results suggest that the electrostatic interactions between the anions and cationic framework are not dominant in the Co-tpy, which is consistent with the selectivity between halides.

### Selectivity for ReO_4_^–^

ReO_4_^–^ is considered a surrogate for ^99^TcO_4_^–^, which is a common pollutant in radioactive wastewater from nuclear power plants. Consequently, removal of this toxic oxo-anion via anion-exchange technology has recently been the subject of intensive investigation.^52–55^ To investigate the selectivity for ReO_4_^–^, anion-exchange reactions were performed in solutions containing different molar ratios of sulfate and perrhenate (ranging from 1:1 to 1000:1). The resulting selectivity was analyzed by using SEM-EDS (Figure S19). The relative S Kα peak intensity decreased with a decrease in SO_4_^2–^ concentration. Figure 7d shows that the selectivity for ReO_4_^–^ over SO_4_^2–^ was 80% at the equivalent molar ratio (5 μM), indicating that Co-tpy exhibits a higher selectivity for ReO_4_^–^ than SO_4_^2–^. An increasing SO_4_^2–^ molar ratio resulted in a corresponding decrease in ReO_4_^–^ selectivity, reaching a minimum of 36% under a 1000:1 molar ratio (5 mM SO_4_^2–^ and 5 μM ReO_4_^–^). Therefore, Co-tpy can capture ReO_4_^–^ from H_2_O containing competing anions.

Selective anion-exchange was also investigated using other environmentally toxic metal oxo-anions such as Cr_2_O_7_^2–^ and MnO_4_^–^. Figure S20 shows the SEM-EDS spectra of Co-tpy after the anion-exchange reaction in a mixed solution of metal oxo-anions (5 μM) and the competitive SO_4_^2–^ anion (5 mM). The selectivity of MnO_4_^–^ and ReO_4_^–^ exhibited an equivalent selectivity of 36% under the equivalent conditions. In contrast, the SEM-EDS spectra following the SO_4_^2–^ and Cr_2_O_7_^2–^ reaction contained no detectable peaks corresponding to the oxo-anions (Figure S20b), indicating low selectivity for Cr_2_O_7_^2–^. The higher selectivity toward ReO_4_^–^ and MnO_4_^–^ stems from their lower charge density compared to that of Cr_2_O_7_^2–^. Therefore, Co-tpy exhibits high selectivity toward group 7 metal oxo-anions.

### Selectivity for Acid Red 91

The irreversibility of anion-exchange from AR91^2–^ to SO_4_^2–^ indicated high selectivity for AR91^2–^ over SO_4_^2–^. The anion-exchange selectivity was investigated by using a mixed solution containing SO_4_^2–^ and AR91^2–^. The selectivity was analyzed by using SEM-EDS (Figure S21). The selectivity for AR91^2–^ was analyzed from the S Kα peak area. As shown in Figure 7e, the 1000:1 SO_4_^2–^:AR91^2–^ solution yielded 95% AR91^2–^ selectivity. Surprisingly, even the 100 000:1 SO_4_^2–^:AR91^2–^ solution (100 mM and 1 μM, respectively) yielded >80% selectivity, indicating the notable selectivity toward xanthene dye anions. Fe-tpy also exhibited selectivity toward organic dye anions. The SEM-EDS spectrum in Figure S22 shows that Fe-tpy selectively captured AR91^2–^ from a concentrated Na_2_SO_4_ aqueous solution at a molar ratio of SO_4_^2–^:AR91^2–^ = 1000:1.

The selectivity toward other organic dye anions was also investigated. Owing to the presence of sulfonate groups in dye anions, mixed solutions containing the dye anions (5 μM) and BF_4_^–^ (5 mM) were used. The SEM-EDS spectra in Figures 7f and S23 show that the selectivities toward IC^2–^, TO^–^, BPB^–^, BCG^–^, and ET^–^ were 97%, 79%, 68%, 57%, and 43%, respectively. Therefore, Co-tpy exhibits a high selectivity in anion-exchange reactions with other organic dyes.

The high selectivity toward the organic dyes was demonstrated by extracting AR91^2–^ from artificial seawater. A Co-tpy film was immersed for 3 days in a 5 μM AR91^2–^ solution prepared from artificial seawater, which contained 465 mM NaCl, 1.5 mM K_2_CO_3_, 3.3 mM K_2_SO_4_, 40 mM MgCl_2_, and 14 mM MgSO_4_.^56^ The postreaction SEM/EDS spectrum exhibited an intense Cl Kα peak and a weak Br Kα peak at 11.9 keV (Figure 8). XPS analyses also revealed the presence of bromine (Figure S24). The artificial seawater does not contain bromide salts; therefore, the detected bromine is attributed to anion exchange with AR91^2–^, which contains two Br atoms per anion (Figure 4a). The Co-tpy film immersed in artificial seawater without AR91^2–^ did not exhibit a Br Kα peak (Figure S25). The AR91^2–^ content in the Co-tpy film was quantified at approximately 5% of the total negative charge. Given that the molar ratio of AR91^2–^ in the artificial seawater solution was <2 ppm, the successful detection of AR91^2–^ in the Co-tpy film demonstrated the high potential as anion-exchange membranes for water remediation.

**Figure 8:**
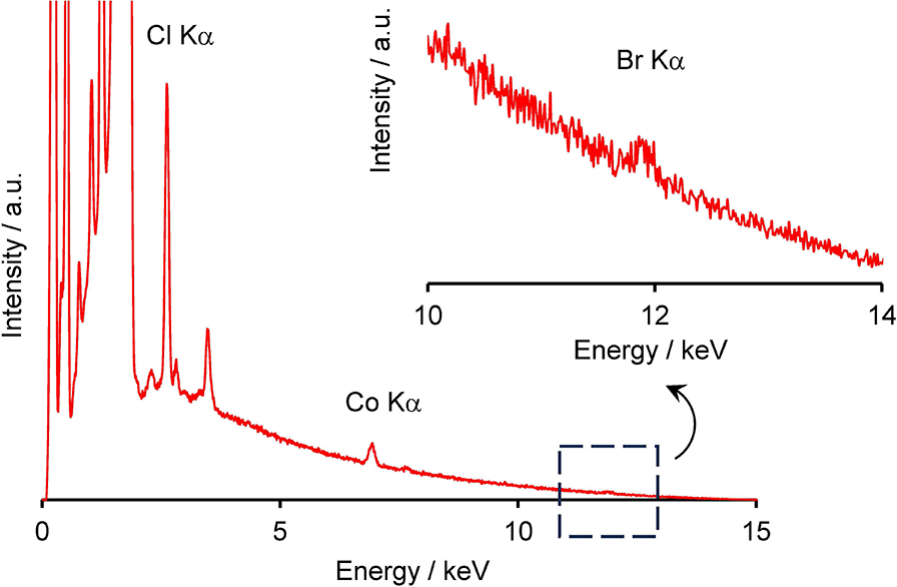
AR91^2–^ extraction from artificial seawater using Co-tpy (2Cl^–^). SEM-EDS spectrum of Co-tpy (2Cl^–^) immersed in artificial seawater containing 5 μM AR91^2–^. The inset shows a magnification of the Br Kα peak region.

### Mechanistic Insights into Selectivity

Owing to the low crystallinity of Co-tpy,^31^ the exact driving force for the selective anion-exchange is difficult to determine experimentally. Instead, the experimental selectivity results provide mechanistic insights into the anion-exchange phenomena. The selectivity for halides described in the previous sections indicates that the electrostatic interactions are not dominant in the anion-exchange reaction. Therefore, the plausible mechanism of selective anion-exchange involves more favorable supramolecular interactions between the anions and the cationic framework of Co-tpy. The soft nature of iodide allows it to avoid repulsive anion−π interactions between the tris­(terpyridine) ligand and the halide. Polyatomic oxo-anions can interact with cationic frameworks at multiple points, resulting in interactions that are energetically more favorable than those involving single-atomic halides. Organic dye anions can also interact with the framework through multiple interactions through their developed π-conjugated moieties such as C–H···π, π–π, and electrostatic interactions. These enhanced interactions result in high selectivity in the anion-exchange reaction.

Selectivity in anion exchange is determined by a balance of multiple supramolecular interactions. Experiments using a ternary mixture of SO_4_^2–^, ReO_4_^–^, and AR91^2–^ (1.67 mM each) showed preferential incorporation of ReO_4_^–^ (Figure S26), which differs from the pairwise selectivity trends (Figure 7d,e). These results suggest that the selectivity mechanism is governed not by a single type of interaction but by the interplay of multiple supramolecular interactions.

### Electrochemistry of Co-tpy Including Dye Anions

Bis­(terpyridine)­cobalt­(II) complexes are characterized by their redox activity. The redox reaction of ionic polymers typically involves insertion or extraction of ions.^57–59^ Therefore, the electrochemical release of organic dye anions was assessed by investigating the electrochemistry of Co-tpy (2BCG^–^) on F-doped tin oxide (FTO) substrates under an Ar atmosphere. Figure 9a shows the cyclic voltammogram of Co-tpy (BCG^–^) in 0.1 M *n*Bu_4_NPF_6_/CH_3_CN. The voltammogram shows that the chemically reversible reduction occurred at approximately −1.15 V vs Fc^+^/Fc, which is consistent with the redox potential of the [Co­(tpy)_2_]^2+^/[Co­(tpy)_2_]^+^ redox couple.^25^ If the reduction process involved the extraction of BCG^–^ from the polymer, the color of Co-tpy (2BCG^–^) would gradually bleach due to the replacement of BCG^–^ with PF_6_^–^ during the redox cycles. However, the color of the Co-tpy (2BCG^–^) film remained unchanged even after 10 redox cycles (Figure 9b). Moreover, the electrolyte exhibited faint colorization after redox cycling, further confirming minimal leaching of BCG^–^ (Figure S27). UV–vis spectroscopy (Figure 9c) determined the amount of leached BCG^–^ to be 1.3 × 10^–9^ mol. The mole of electrons related to the redox reaction was calculated to be 2.9 × 10^–8^ mol, as quantified by the Co-tpy (2BCG^–^) redox peak area. Therefore, the BCG^–^ leaching ratio was only 2.2% after 10 redox cycles, which is remarkably lower than the theoretical value of 99.9% expected if each one-electron reduction cycle accompanied the random extraction of half of the BCG^–^ anions. Raman spectroscopy and SEM-EDS confirmed the irreversibility and framework stability. The Raman and SEM-EDS spectra after 10 redox cycles were essentially identical with those of the initial state (Figure S28a,b). SEM images also showed no significant change in film morphology after electrochemical cycling (Figure S28c,d), confirming the stability of Co-tpy (2BCG^–^) under redox cycles. AR91^2–^ also exhibited a low leaching ratio of 25% after 10 redox cycles (Figure S29). The leaching ratio of AR91^2–^ is higher than that of Co-tpy (2BCG^–^) owing to the slightly irreversible redox process of Co-tpy (AR91^2–^), as indicated by the decreasing peak intensity during redox cycling (Figure S30). However, this is still lower than the theoretical ratio based on random electrochemical anion-exchange. These results suggest that the organic dye anions in Co-tpy were neither electrochemically nor spontaneously exchangeable, indicating a high selectivity for organic dye anions to realize irreversible anion-exchange reactions. Such a low leaching ratio will lead to new electrochemical applications of organic dye anion-containing polymer thin films in condensed electrolytes.

**Figure 9:**
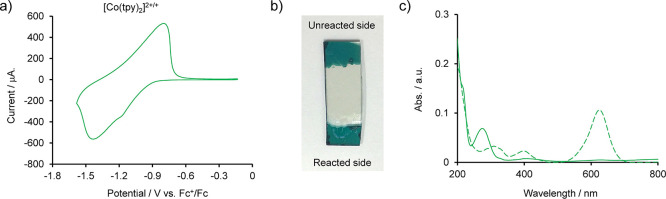
Electrochemistry of Co-tpy (2BCG^–^). (a) Cyclic voltammogram of Co-tpy (2BCG^–^) in 0.1 M *n*Bu_4_NPF_6_/CH_3_CN (scan rate: 50 mV/s). (b) Photographic image of the Co-tpy (2BCG^–^) film on an FTO substrate. The bottom side was subjected to redox cycling, while the top side was not. (c) UV–vis spectra of the electrolyte solution after the 10 redox cycles (solid line) and 2.8 μM NaBCG/CH_3_CN solution (dotted line).

## Conclusion

In summary, we demonstrated that chloride ions in bis­(terpyridine)­cobalt­(II) coordination polymer thin films can be efficiently and selectively exchanged with a broad range of anions, including inorganic anions, organic dyes, and metal oxo-anions. Notably, oxo-anions and anionic organic dyes were preferentially incorporated into the Co-tpy framework even in the presence of high concentrations of competing inorganic anions, highlighting the exceptional selectivity of this system. Such wide applicability and robustness in anion-exchange behavior make these films particularly attractive membranes for environmental sensing and water remediation, including the selective removal of hazardous anions from seawater. Importantly, coordination polymers constructed from structurally simple terpyridine ligands already exhibit high anion selectivity, underscoring the intrinsic potential of this molecular design. Moreover, the introduction of tailored functional groups onto the terpyridine ligands is expected to further enhance the anion-recognition capability and accelerate exchange kinetics, enabling the rapid and efficient removal of toxic anions. In addition, the organic dye anions incorporated into the Co-tpy framework showed negligible leaching during redox cycling of the polymer, demonstrating the stability of the anion-storage process. Collectively, this study establishes bis­(terpyridine)­metal­(II) coordination polymer thin films as a versatile platform for anion-exchange and storage and further highlights their promise as redox-active, anion-exchanged thin films for future electrochemical and environmental applications.

## Experimental Section

### Materials

Co-tpy (2Cl^–^) and Fe-tpy (2BF_4_^–^) were prepared by the liquid–liquid interfacial coordination method.^25,27^ Co-tpy (2NO_3_^–^), Co-tpy (2BF_4_^–^), and Ni-tpy (2Cl^–^) were synthesized by using the same liquid–liquid interfacial coordination protocols. All other chemicals were commercially available and used without further purification. Artificial seawater was prepared according to the previously reported literature.^56^ Water was purified using Autopure WD500 (Yamato Scientific Co., Ltd.) and Merck Milli-Q SQ2 systems. Organic solvents used in this study were purchased in HPLC grade and used without further purification.

### Preparation of Co-tpy (2Cl^–^)^27^

In a 50 mL glass vial (ca. 4 cm in diameter), 10 mL of H_2_O was layered on a 0.1 mM terpyridine ligand solution in CH_2_Cl_2_ (10 mL). To the aqueous phase, 50 mM aqueous CoCl_2_ (10 mL) was gently added by slow pipetting. The reaction container was kept calm for 3 days to form an orange film at the H_2_O/CH_2_Cl_2_ interface. The aqueous phase was diluted to less than 0.1 mM by repeating removal of two-thirds of the aqueous phase and addition of pure water to the remaining aqueous phase. Then, pure ethanol was layered on the aqueous phase, and all solvents were removed from the bottom of reaction container. The remaining Co-tpy (2Cl^–^) film was dispersed and preserved in ethanol. The Co-tpy (2Cl^–^) film was deposited on various substrates by dropcast of the dispersion.

### General Anion-Exchange Reaction Procedure

An ethanolic suspension of Co-tpy (2Cl^–^) flakes was deposited on substrates such as silicon, quartz, glass, and graphite paper substrates. The decorated substrates were placed at the bottom of a 20 mL glass vial and immersed in 5 mL of aqueous solutions containing 5 mM sodium salts of the anions for 3 days. After immersion, the substrate was washed thrice with pure water and ethanol and dried under aerobic conditions.

### Apparatus

#### AFM

AFM measurement was carried out using an Agilent Technologies 5500 Scanning Probe Microscope with a PPP-NCL or NCH silicon cantilever (Nano World) and a NaioAFM (Nanosurf AG) with a PPP-NCLR silicon cantilever (Nano World) in high-amplitude mode (Tapping Mode) under ambient conditions. Co-tpy (*n*X^*m*–^) on Si substrate was used for the measurements.

#### SEM-EDS

SEM-EDS was performed using a JEOL-7000 Neoscope scanning electron microscope equipped with an EDS analyzer. The SEM-EDS analyses were performed using an electron beam with a 15 kV acceleration voltage. Co-tpy (*n*X^*m*–^) on a Si substrate was used for the measurements. The selectivity of the anion-exchange reaction was determined from the peak area of representative peaks of the anions in which the peak intensities were normalized using the intensity of the Co Kα peak.

#### XPS

XPS was performed using an Ulvac Phi VersaProbe 5000 and VersaProbeIII spectrometer with Al Kα radiation (25 W, 15 kV). The binding energy of XPS was calibrated using the C 1s peak at 284.6 eV. Co-tpy (*n*X^*m*–^) on graphitic carbon paper substrate was used for the measurements.

#### UV–vis Spectroscopy

UV–vis spectra were obtained by using a JASCO V770 spectrometer using the transmittance method. Co-tpy (*n*X^*m*–^) on a quartz substrate was used for the measurements. The time-dependent measurements were conducted using Co-tpy (2Cl^–^) on a Si substrate (∼8 mm × 8 mm) placed at the bottom of the quartz cell (optical length of 1 cm). A 3 μM aqueous solution containing acid red 91 or KReO_4_ (3 mL) was added to the cell, and UV–vis spectra were recorded at certain time intervals.

#### Raman Spectroscopy

Raman spectra were collected by using a JASCO NRS5500 spectrometer with 532 nm laser light irradiation. Co-tpy (*n*X^*m*–^) on a Si substrate was used for the measurements.

#### Electrochemical Measurements

Electrochemical measurements were performed using a custom-made electrochemical cell at a standard three-electrode setup. FTO substrates decorated with Co-tpy (*n*X^*m*–^), Pt wire, and a homemade Ag^+^/Ag electrode (0.01 M AgClO_4_ in 0.1 M *n*Bu_4_NClO_4_/CH_3_CN) were employed as the working, counter, and reference electrodes, respectively. The potential was controlled using an ALS750E electrochemical analyzer. All electrochemical measurements were performed in 0.1 M *n*Bu_4_NPF_6_/CH_3_CN (5 mL) under an Ar atmosphere. The potential was calibrated using the redox potential of Fc^+^/Fc recorded under the same experimental conditions. After the CV measurements, the electrolyte solution was collected and diluted to 10 mL with CH_3_CN, and the UV–vis spectra of the electrolyte were recorded by using a quartz cell with 1 cm optical length.

## Supplementary Material



## Notes

Additional characterization data for the anion-exchanged M-tpys, selectivity analyses data, and electrochemical measurements (SEM-EDS, Raman spectra, XPS, UV–vis spectra, AFM images, photographic images, and cyclic voltammograms) (PDF)
