# Supplementary material for: Highly Selective and Irreversible Anion-Exchange in Two-Dimensional Bis(terpyridine)metal(II) Polymer Thin Films
Source: ACS Appl Mater Interfaces. 2026 Mar 31;18(14):20922–32. doi: 10.1021/acsami.6c00601 (PMC13088033; doi:10.1021/acsami.6c00601)
Supplement: Supplementary file 1 [file am6c00601_si_001.pdf]

Supporting Information

Highly Selective and Irreversible Anion-Exchange in Two-Dimensional Bis(terpyridine)metal(II) Polymer Thin Films

Kenji Takada*, Hiroshi Nishihara*

Research Institute for Science and Technology, Tokyo University of Science, 2641 Yamazaki, Noda, Chiba 278-8510 Japan

*Corresponding Authors

Kenji Takada: takada.k.ag@rs.tus.ac.jp, Hiroshi Nishihara: nisihara@rs.tus.ac.jp

Index

A. Characterization of anion-exchange reaction of Co-tpy with halides
B. Supporting data for anion-exchange reaction of Co-tpy with inorganic anions
C. Anion-exchange reaction from acid red 91 to sulfate
D. Supporting data for anion-exchange reaction of Co-tpy with metal oxo-anions
E. Kinetic analysis of anion-exchange reaction
F. Anion-exchange reaction of other M-tpys (M = Fe and Ni)
G. Selectivity in anion-exchange reaction of M-tpys
H. Supporting characterization data for acid red 91 extraction from artificial seawater
I. Supporting characterization data for anion-exchange with the mixed-anion solution.
J. Electrochemistry of Co-tpy including organic dye anions

A. Characterization of anion-exchange reaction of Co-tpy with halides

A-1. SEM-EDS elemental mappings

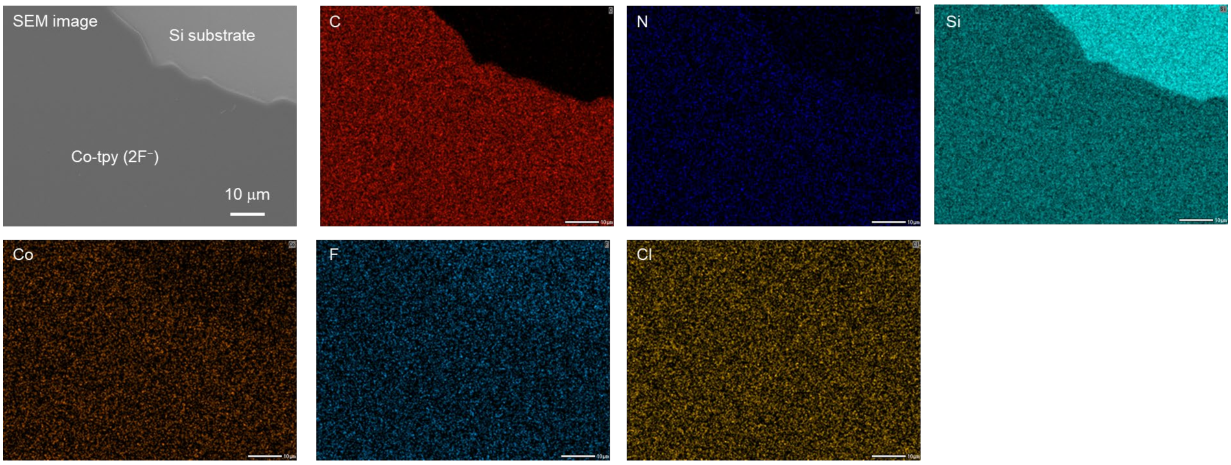

Figure S1. SEM-EDS elemental mapping of anion-exchanged Co-tpy with F^-.

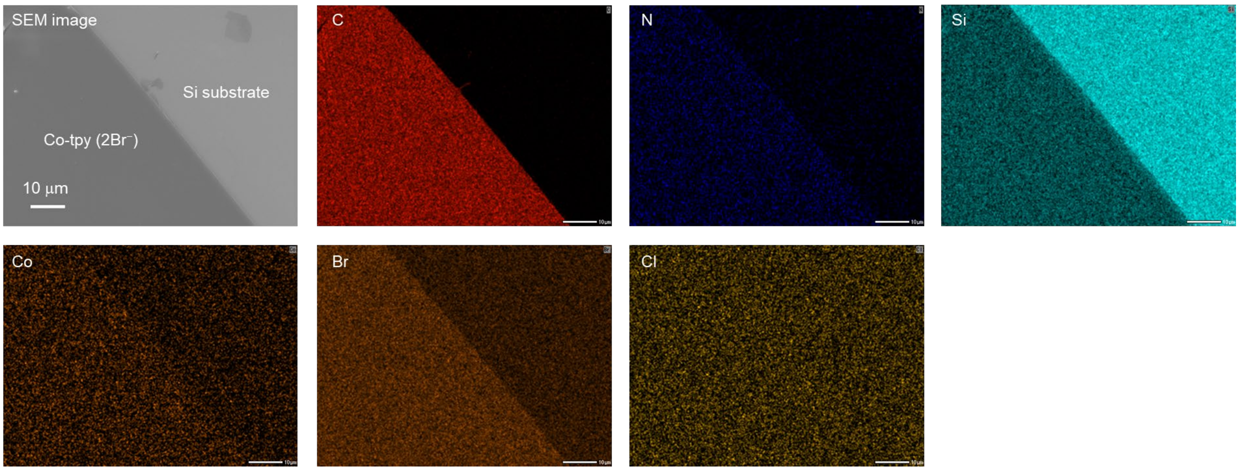

Figure S2. SEM-EDS elemental mapping of anion-exchanged Co-tpy with Br^-.

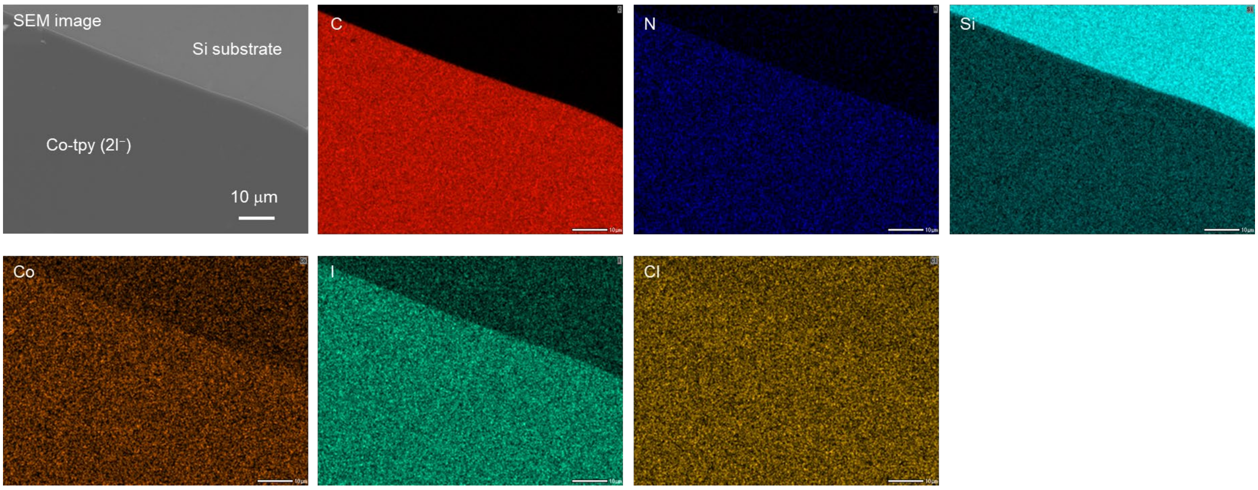

Figure S3. SEM-EDS elemental mapping of anion-exchanged Co-tpy with I^-.

A-2. XPS

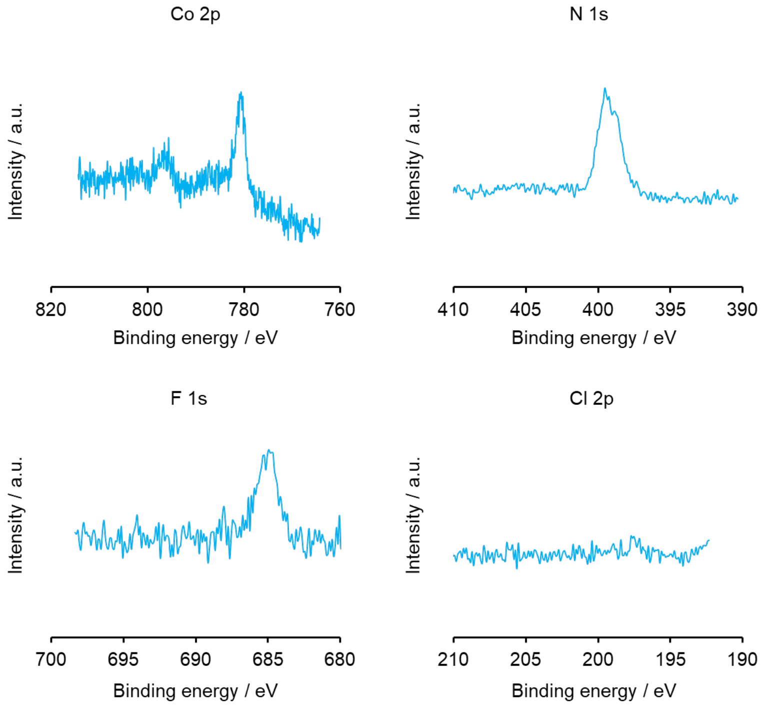

Figure S4. XPS of anion-exchanged Co-tpy with F^- focusing on the core levels of each element.

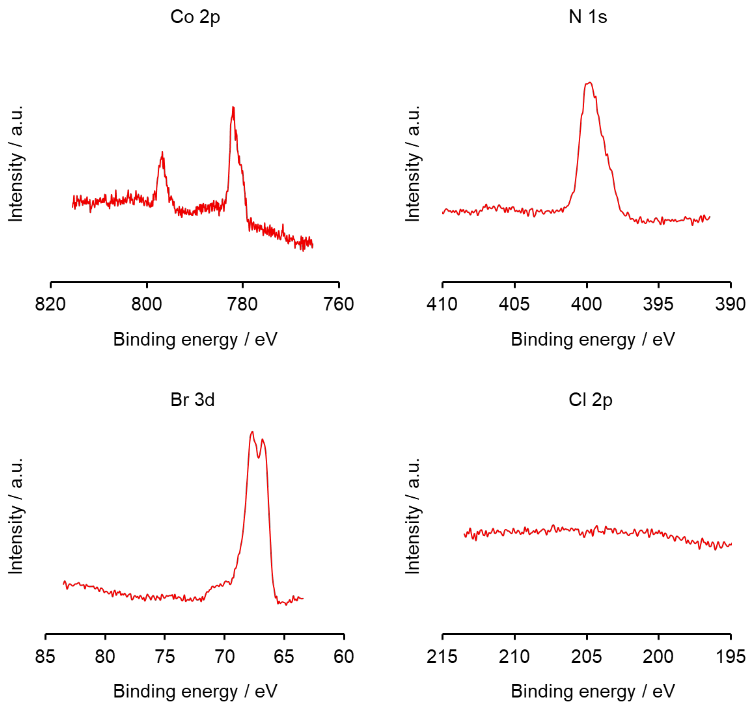

Figure S5. XPS of anion-exchanged Co-tpy with Br^- focusing on the core levels of each element.

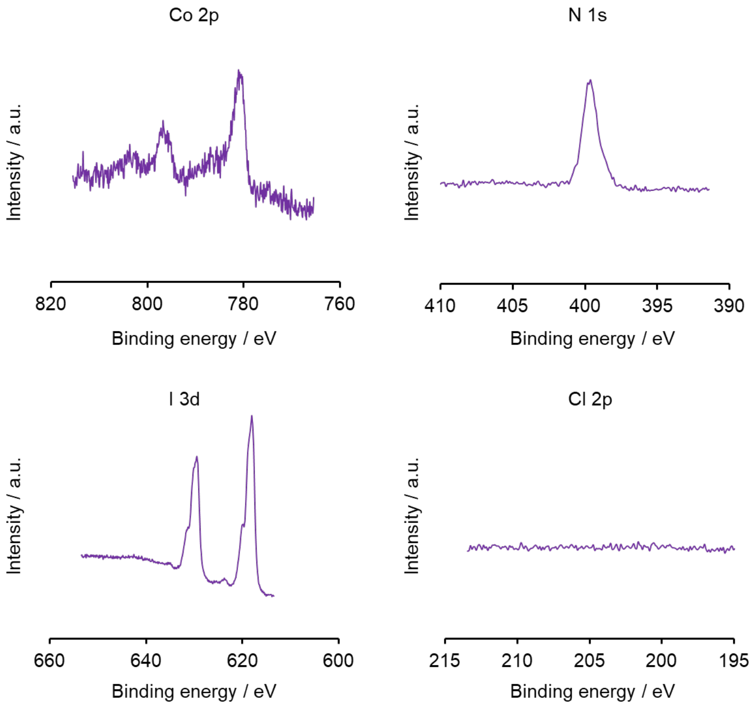

Figure S6. XPS of anion-exchanged Co-tpy with I^- focusing on the core levels of each element.

B. Supporting data for anion-exchange reaction of Co-tpy with inorganic anions

B-1 Electrochemistry of the anion-exchanged Co-tpy

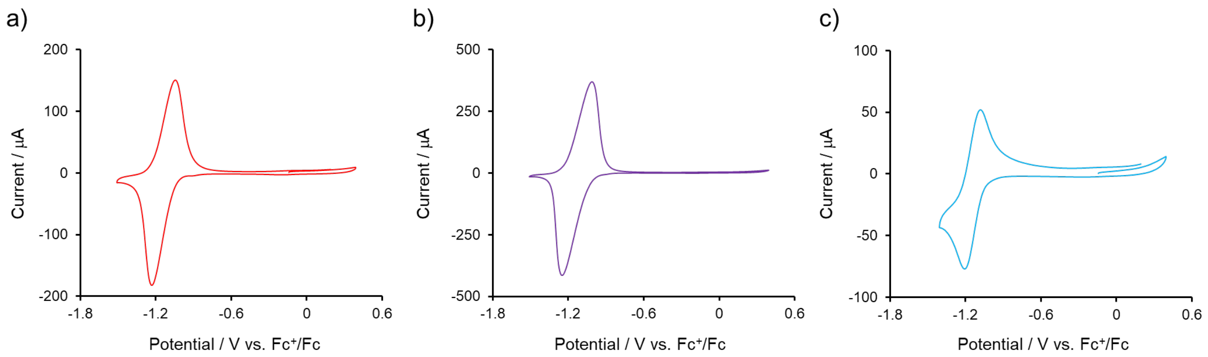

Figure S7. Cyclic voltammetry of (a) Co-tpy ($2Br^-$), (b) Co-tpy ($2NO_3^-$), and (c) Co-tpy ($2CH_3COO^-$). (scan rate: 50 mV/s, 0.1 M *n*Bu^4NPF_6/CH_3CN).

B-2 Cation dependence of the anion-exchange reaction

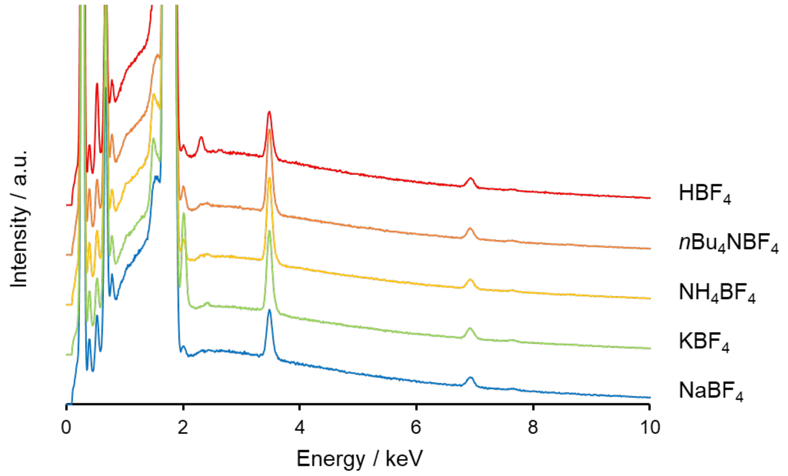

Figure S8. SEM-EDS of anion-exchanged Co-tpy with BF_4^- with different cations.

B-3 AFM analysis of Co-tpy ($2NO_3^-$)

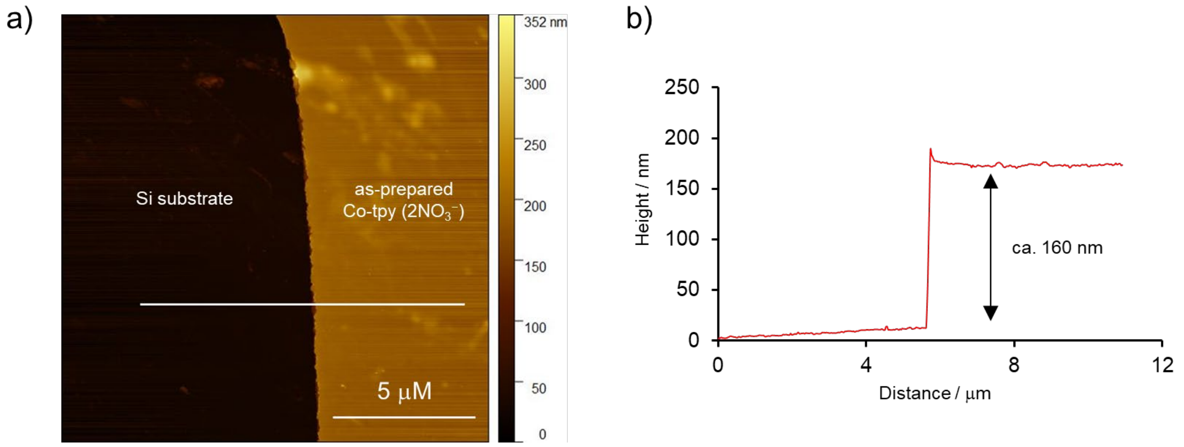

Figure S9. (a) AFM image of Co-tpy ($2NO_3^-$) directly prepared by the liquid-liquid interfacial synthesis. (b) Height profile along the white line in the AFM image (a).

B-4 Inverse anion-exchange reaction from BF_4^- to Cl^-

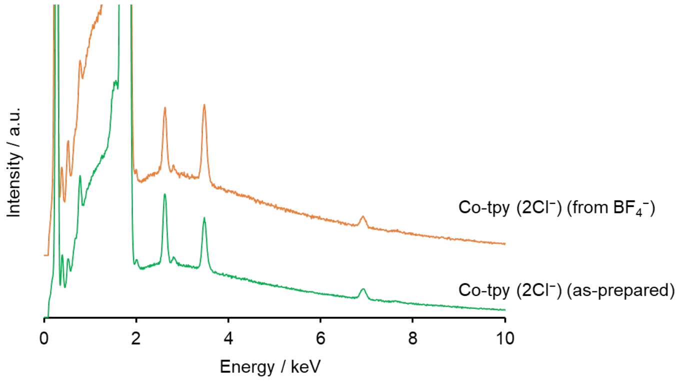

Figure S10. SEM-EDS of Co-tpy ($2Cl^-$) synthesized by anion-exchange from BF_4^- (a) and as-prepared (b).

C.	Anion-exchange reaction from acid red 91 to sulfate

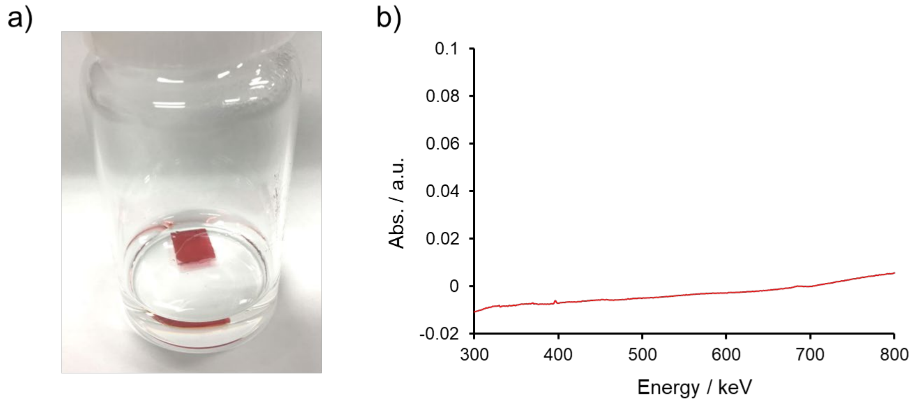

Figure S11. (a) Photograph of anion-exchange reaction of Co-tpy ($AR91^{2-}$) film on glass substrate with SO_4^{2-} after 3 months. (b) UV-vis spectrum of the reaction solution in (a).

D. Supporting data for anion-exchange reaction of Co-tpy with metal oxo-anions

D-1 SEM-EDS spectra of the anion-exchanged Co-tpy with metal oxo-anions using diluted solutions

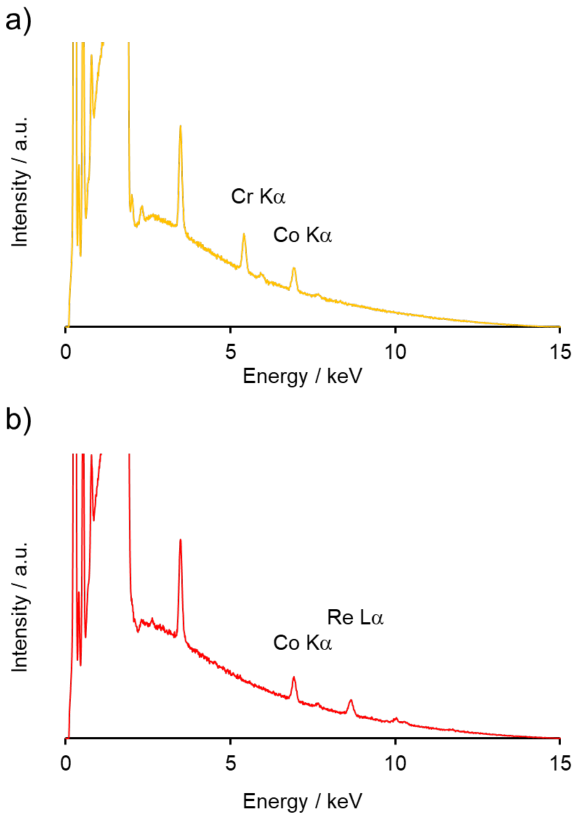

Figure S12. SEM-EDS spectra of Co-tpy film anion-exchanged with 5 μM $Cr_2O_7^{2-}$ (a) and ReO_4^- (b) solutions.

D-2 Electrochemistry of the anion-exchanged Co-tpy with metal oxo-anions

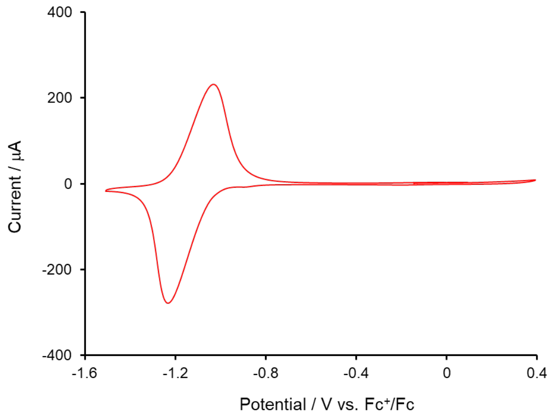

Figure S13. Cyclic voltammetry of Co-tpy ($2ReO_4^-$). (scan rate: 50 mV/s, 0.1 M *n*Bu_4NPF_6/CH_3CN)

E. Kinetic analysis of anion-exchange reaction

E-1 UV-vis spectroscopy

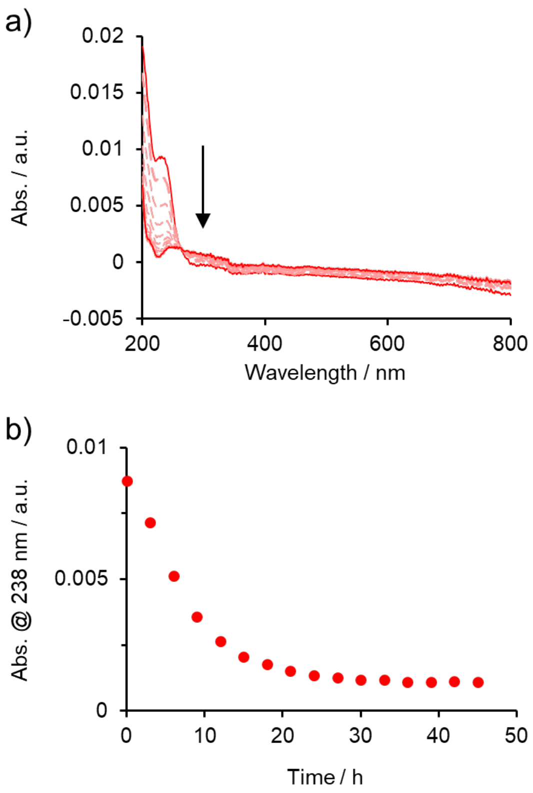

Figure S14. Kinetics of anion-exchange reaction with ReO_4^-. (a) UV-vis spectra. (b) Time-dependent absorptivity at 238 nm.

E-2 Kinetic Analysis

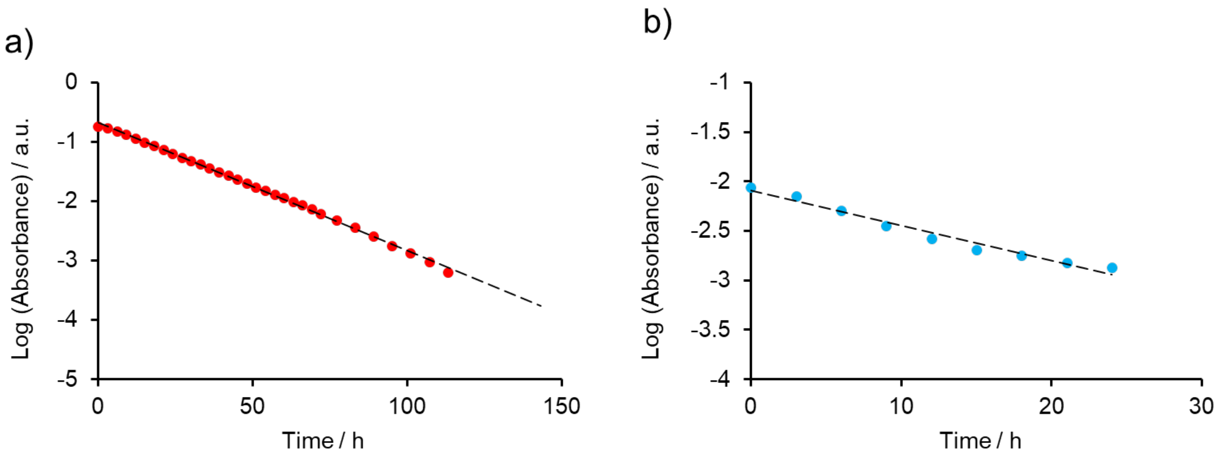

Figure S15. Logarithm plot of the absorbance in the UV-vis spectroscopy during the anion-exchange reaction of Co-tpy with $AR91^{2-}$ at 517 nm (a) and ReO_4^- at 238 nm (b). The dotted lines were linear fitting of experimental plots.

F. Anion-exchange reaction of other M-tpys (M = Fe and Ni)

F-1 Anion-exchange reaction of Fe-tpy

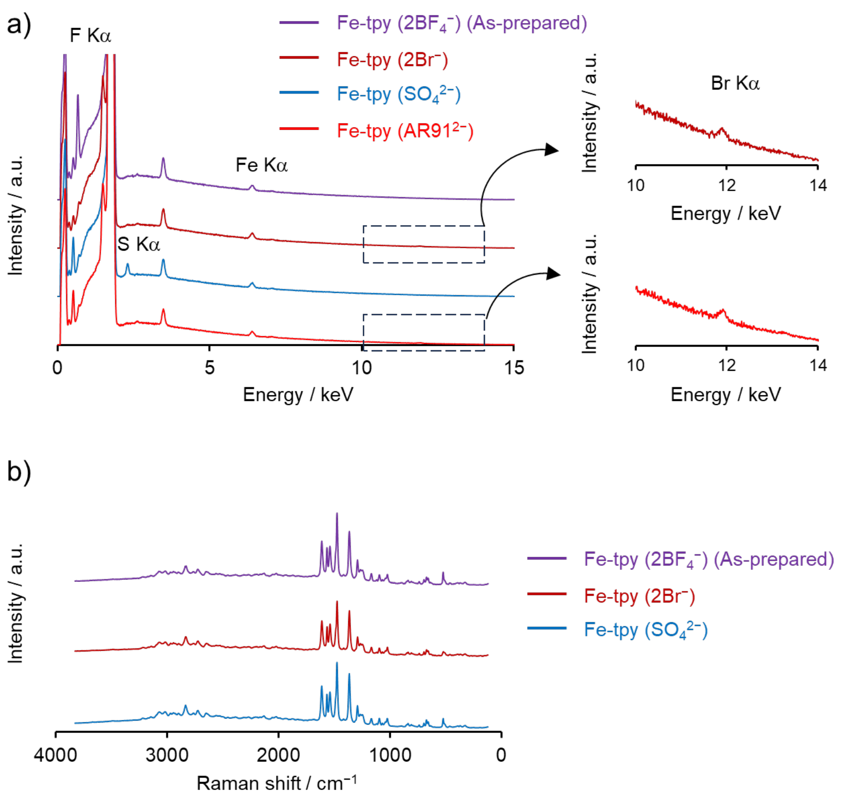

Figure S16. Anion-exchange reaction of Fe-tpy ($2BF_4^-$). (a) SEM-EDS and (b) Raman spectra of as-prepared and anion-exchanged Fe-tpy.

F-2 Anion-exchange reaction of Ni-tpy (2Cl⁻)

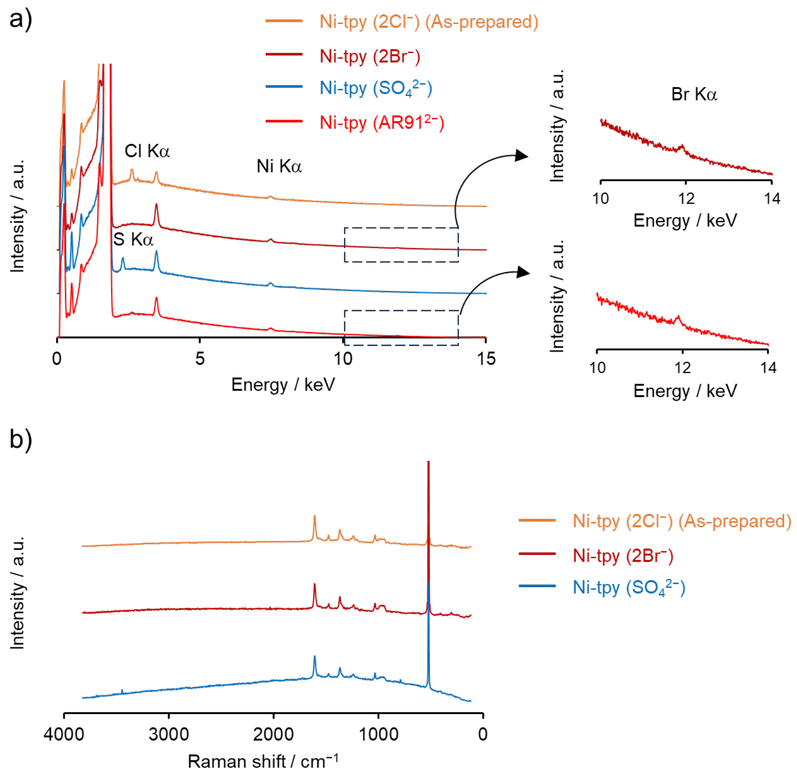

Figure S17. Anion-exchange reaction of Ni-tpy (2Cl⁻). (a) SEM-EDS and (b) Raman spectra of as-prepared and anion-exchanged Ni-tpy.

G. Selectivity in anion-exchange reaction of M-tpys

G-1 Selectivity between BF_4^- and SO_4^{2-}

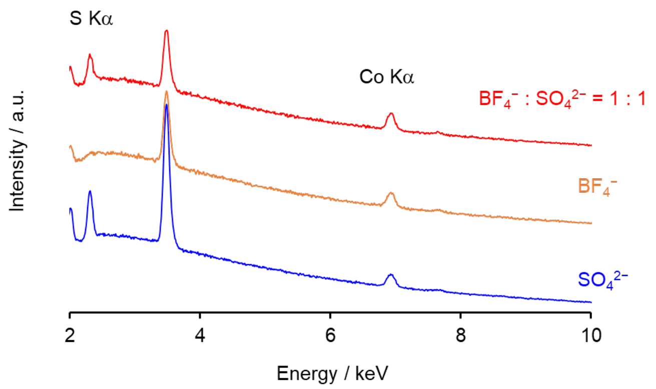

Figure S18. SEM-EDS of anion-exchanged Co-tpy with mixture of BF_4^- and SO_4^{2-} (1:1), BF_4^-, and SO_4^{2-} solutions (2.5 mM for each).

G-2 Selectivity between ReO_4^- and SO_4^{2-}

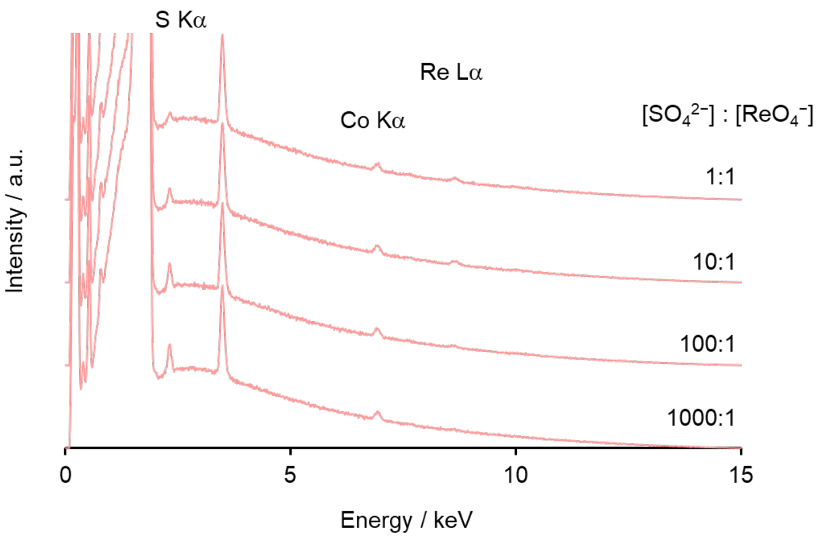

Figure S19. SEM-EDS of Co-tpy after anion-exchange reaction with the mixture of Na_2SO_4 and $KReO_4$ with various molar ratio. ($[ReO_4^-]$ = 5 μM)

G-3 Selectivity for metal oxo-anions

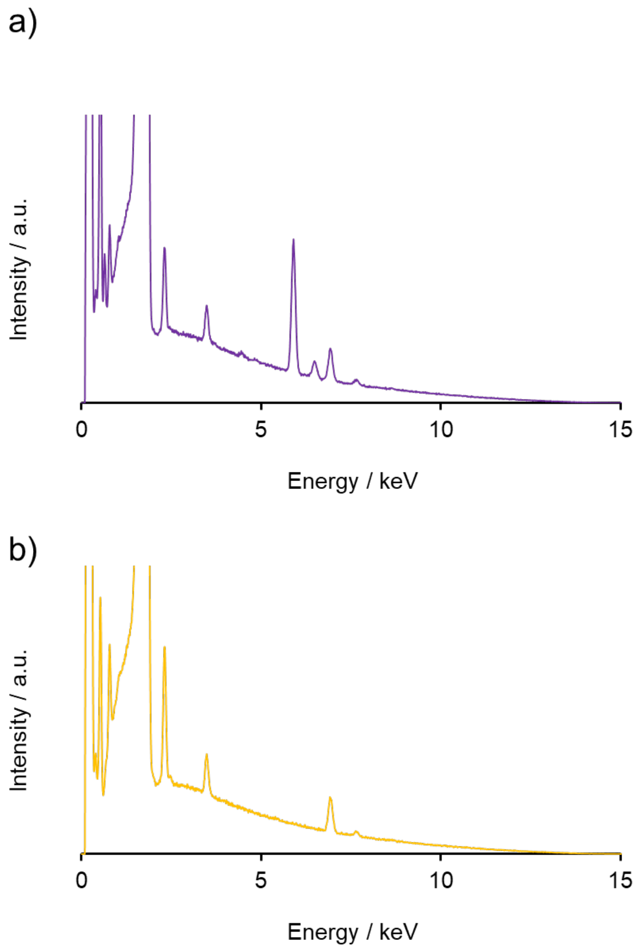

Figure S20. SEM-EDS of Co-tpy after anion-exchange reaction with the mixture of Na_2SO_4 and $KMnO_4$ (a) and Na_2SO_4 and $K_2Cr_2O_7$ (a) with molar ratio of 1000:1. ($[SO_4^{2-}]$ = 5 mM, $[Cr_2O_7^{2-}]$ or $[MnO_4^{-}]$ = 5 mM)

G-4 Selectivity between $AR91^{2-}$ and SO_4^{2-}

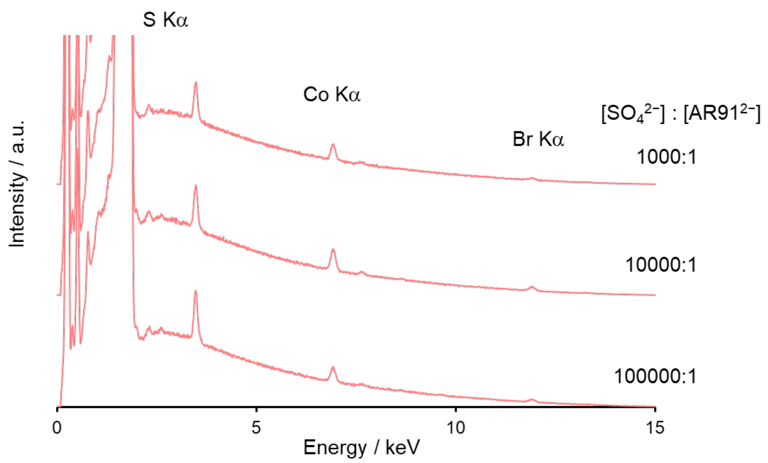

Figure S21. SEM-EDS of Co-tpy after anion-exchange reaction with the mixture of Na_2SO_4 and Na_2AR91 with various molar ratio. ($[AR91^{2-}]$ = 1 μM)

G-5 Selectivity of Fe-tpy between $AR91^{2-}$ and SO_4^{2-}

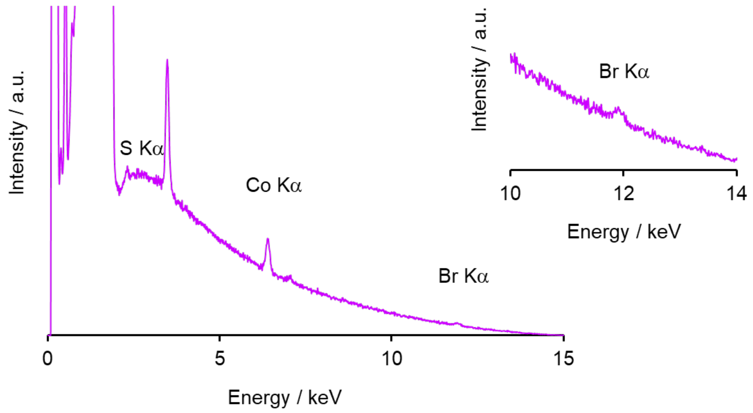

Figure S22. SEM-EDS of Co-tpy after anion-exchange reaction with the mixture of Na_2SO_4 and Na_2AR91 ($[SO_4^{2-}]$ = 5 mM, $[AR91^{2-}]$ = 5 μM)

G-6 Selectivity for other anionic organic dyes

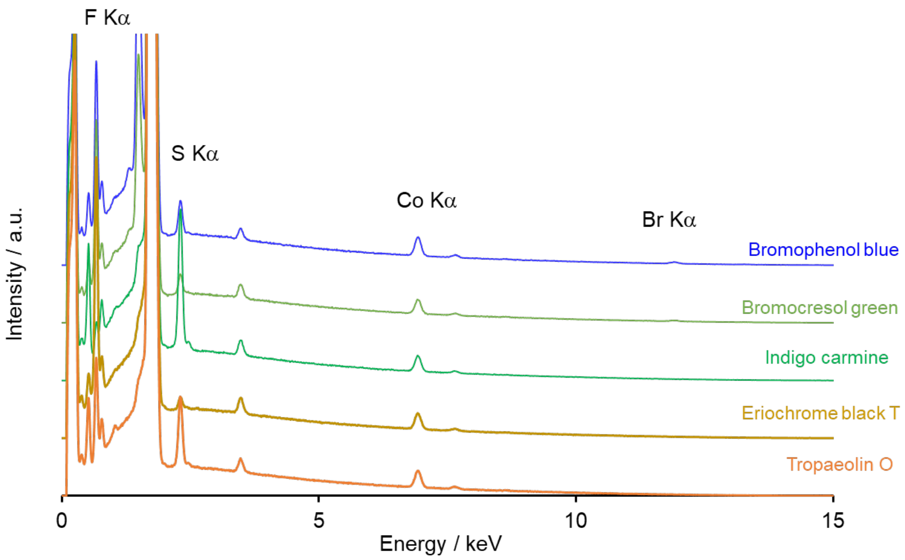

Figure S23. SEM-EDS of Co-tpy after anion-exchange reaction with the mixture of $NaBF_4$ and dyes with various molar ratio. ($[BF_4^-]$ = 5 mM, [dye] = 5 μM)

H. Supporting characterization data for acid red 91 extraction from artificial seawater

H-1 XPS

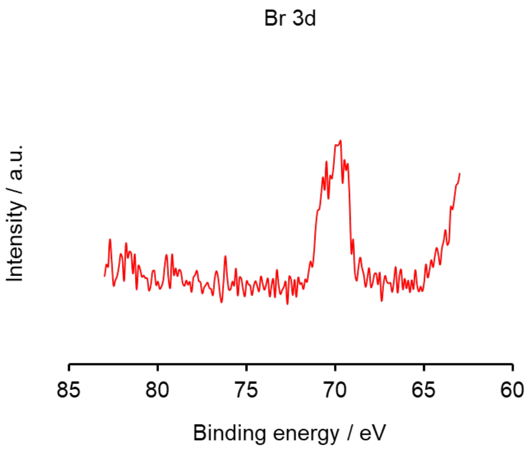

Figure S24. XPS of Co-tpy after the anion-exchange reaction with artificial seawater including $AR91^{2-}$. (Br 3d core level)

H-2 Anion-exchnage reaction with artificial seawater without $AR91^{2-}$

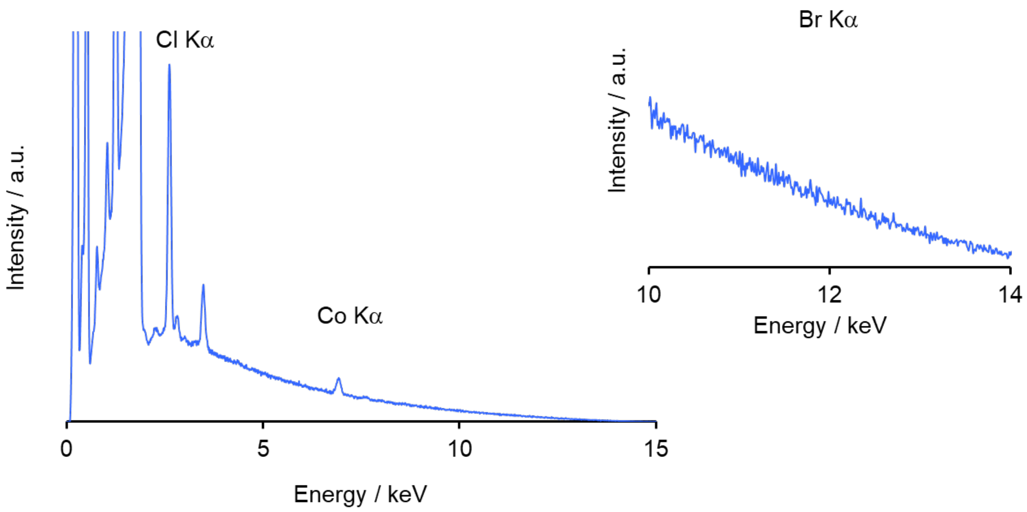

Figure S25. SEM-EDS of Co-tpy after the anion-exchange reaction with artificial seawater not including $AR91^{2-}$.

I. Supporting characterization data for anion-exchange with the mixed-anion solution.

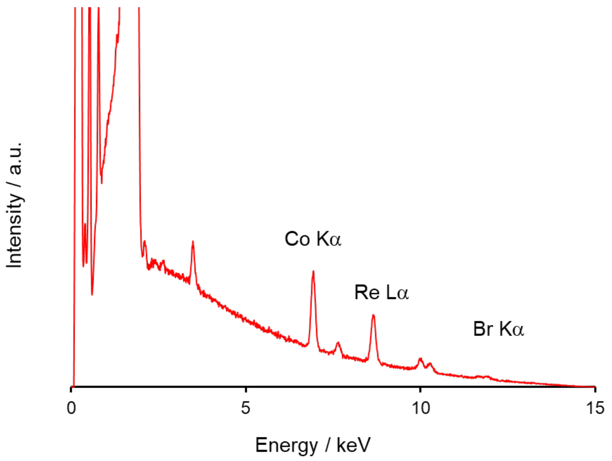

Figure S26. SEM-EDS of Co-tpy after the anion-exchange reaction with SO_4^{2-}, ReO_4^{2-}, $AR91^{2-}$ with equal concentrations (1.67 mM for each).

J. Electrochemistry of Co-tpy including organic dye anions

J-1 Photographs of electrolyte solutions after electrochemical measurements

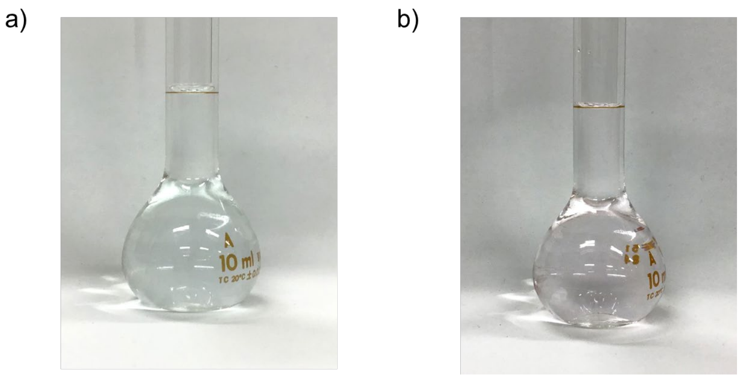

Figure S27. Photographs of electrolyte solution used for electrochemical measurements. (a) Co-tpy ($2BCG^{-}$), (b) Co-tpy ($AR91^{2-}$). Both solutions were diluted to 10 mL.

J-2 Supporting characterization data for Co-tpy ($2BCG^{-}$) before and after the redox cycles

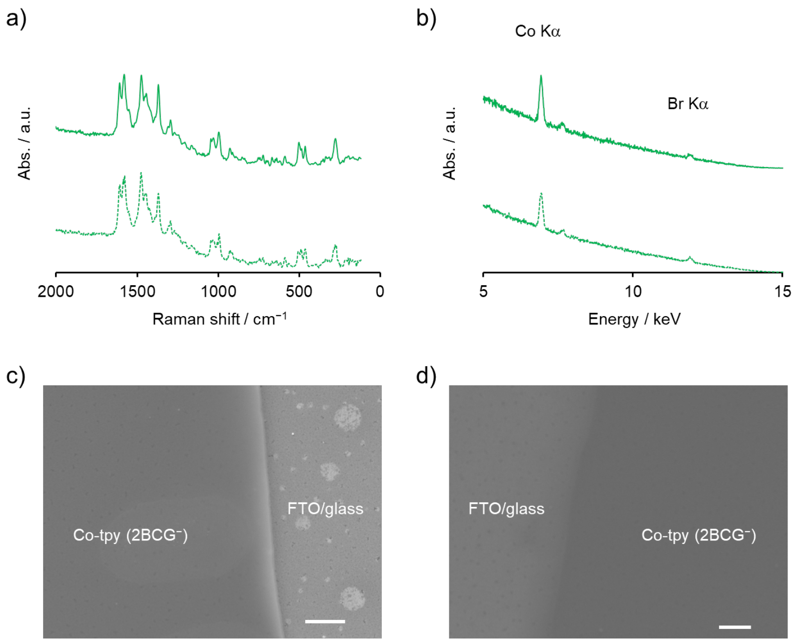

Figure S28. Characterization of Co-tpy ($2BCG^{-}$) before (solid lines) and after (dotted lines) 10 redox cycles. (a) Raman spectra. (b) SEM-EDS spectra. (c) SEM image of Co-tpy ($2BCG^{-}$) on FTO before 10 redox cycles. (d) SEM image of Co-tpy ($2BCG^{-}$) on FTO after 10 redox cycles. (Scale bar: 10 mm)

J-3 Electrochemistry of Co-tpy ($AR91^{2-}$)

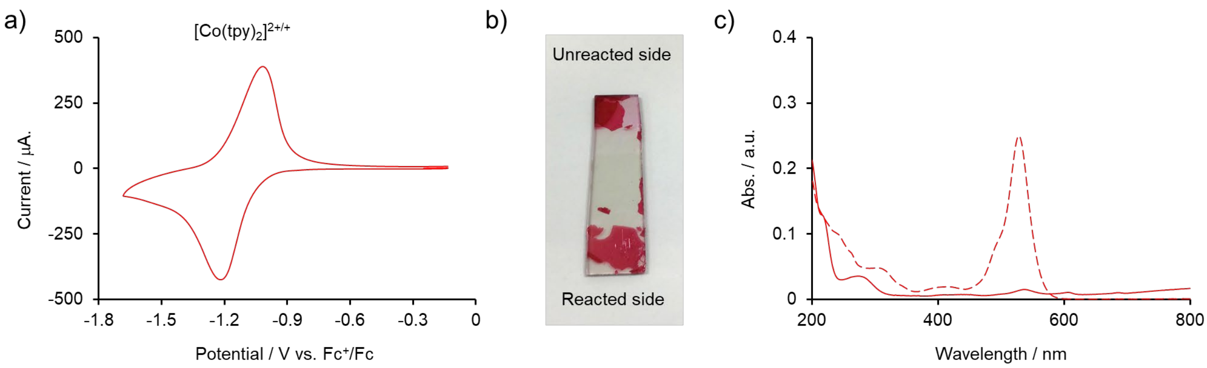

Figure S29. Electrochemistry of Co-tpy ($AR91^{2-}$). (a) Cyclic voltammogram of Co-tpy ($AR91^{2-}$) in 0.1 M $n$$Bu_4NPF_6/CH_3CN$. (scan rate: 50 mV/s) (b) Photograph of Co-tpy ($AR91^{2-}$) on FTO. The bottom side was subjected to the redox cycles while the top side was not. (c) UV-vis spectra of the electrolyte solution after the 10 redox cycles (solid line) and 8.7 µM Na_2AR91/CH_3CN-H_2O (v/v = 49/1) solution (dotted line).

J-4 Durability for the redox cycles

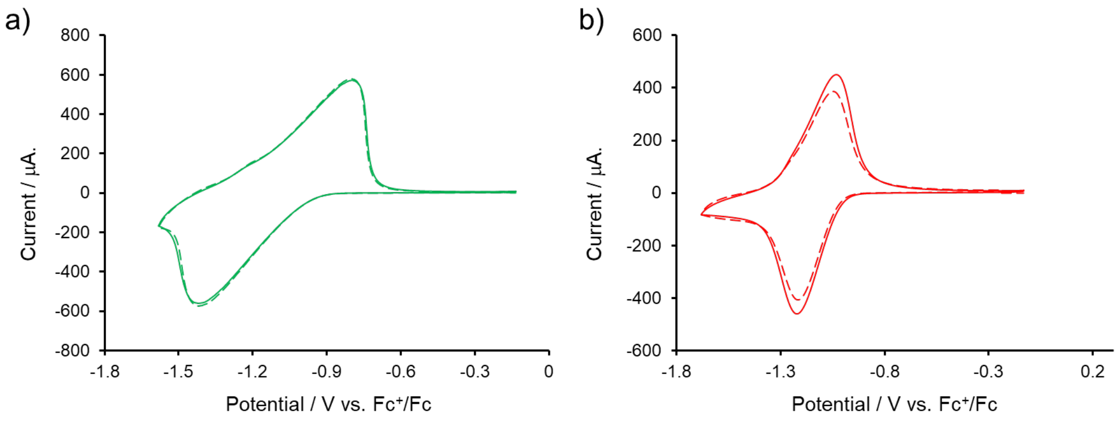

Figure S30. Cyclic voltammetry of Co-tpy ($2BCG^-$) (a) and Co-tpy ($AR91^{2-}$) (b). Solid and dotted lines represent 5th and 10th redox cycles, respectively (0.1 M $n$$Bu_4NPF_6/CH_3CN$, scan rate: 50 mV/s). While voltammograms were constant for Co-tpy ($2BCG^-$), significant decrease in peak intensity was observed for Co-tpy ($AR91^{2-}$), which resulted in the higher leaching ratio.
